# Supplementary material for: Genomic analysis of human-infecting Leptospira borgpetersenii isolates in Sri Lanka: Expanded PF07598 gene family repertoire and less genome reduction than bovine isolates
Source: PLoS Negl Trop Dis. 2026 Mar 27;20(3):e0012540. doi: 10.1371/journal.pntd.0012540 (PMC13046281; doi:10.1371/journal.pntd.0012540)
Supplement: S1 File — (DOCX) [file pntd.0012540.s001.docx]

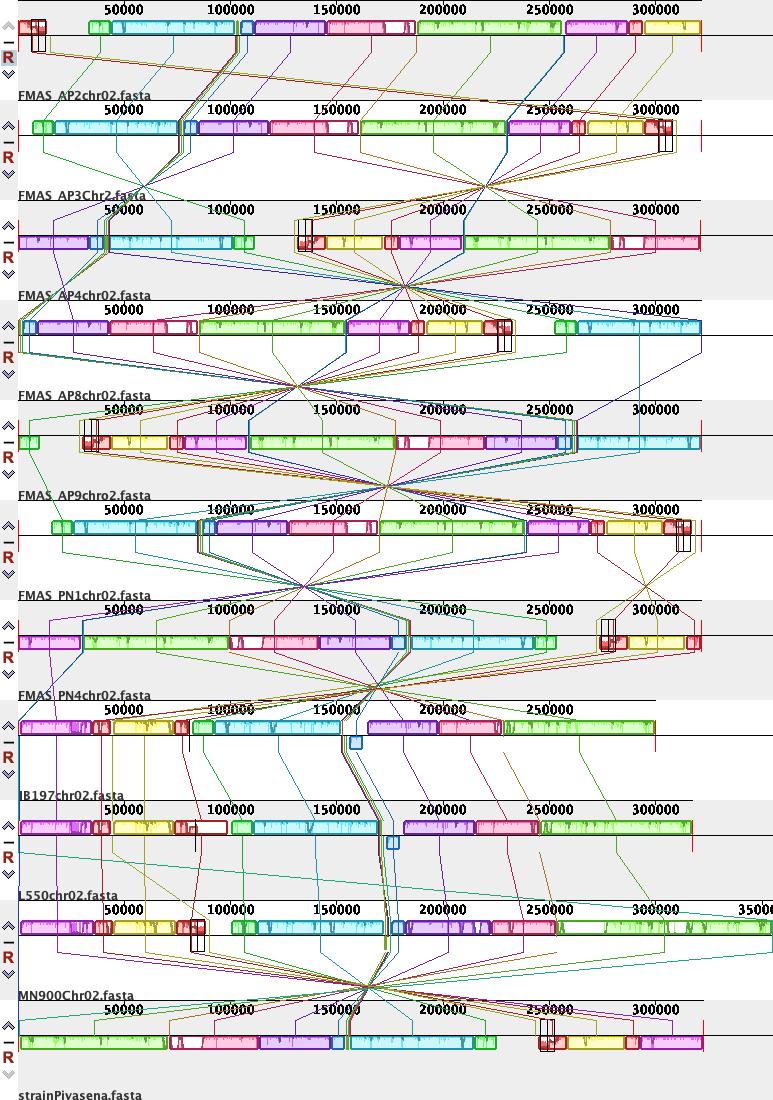


**Supplemental Figure S**1**.** **Genomic alignments of seven -ex human, Sri Lankan *Leptospira borgpetersenii* isolates and comparison with,L550 and JB197 ,MN 900 and strain Piyasena using Mauve 2**

Snap shot comparing the genomic organization of three Sri Lankan *L. borgpetersenii* isolates with other genomes. Genomes of these strains were aligned and arranged using the Mauve genome aligner in the following order: Top, FMAS_AP2, , FMAS_AP3, FMAS_AP4, FMAS_AP8,FMAS_AP9, FMAS_PN1, FMAS_PN4, JB 197, L550 and MN 900; at very botton, , *L. borgpeterseniis* serovar Ceylonica, strain Piyasena. Large collinear blocks (LCBs) correspond mainly to conserved syntenic regions, as represented by colored boxes. The lines between the genomes connect the blocks that are conserved between two strains and larger scale rearrangements
