# Supplementary material for: Genomic analysis of human-infecting Leptospira borgpetersenii isolates in Sri Lanka: Expanded PF07598 gene family repertoire and less genome reduction than bovine isolates
Source: PLoS Negl Trop Dis. 2026 Mar 27;20(3):e0012540. doi: 10.1371/journal.pntd.0012540 (PMC13046281; doi:10.1371/journal.pntd.0012540)
Supplement: S2 File — (DOCX) [file pntd.0012540.s002.docx]

**Supplemental File S2. Amino Acid Sequences LipL32 and Lig B**

>fig|174.58.peg.2326 LipL32 [Leptospira borgpetersenii FMAS_AP3]

MKKLSILAISAALFASITACGAFGGLPSLKSSFVLSESTVPGTNETVKTFLPYGSVINYY

GYVKPGQAPDGLVDGNKKAYYLYVWIPAVIAEMGVRMISPTGEIGEPGDGDLVSDAFKAA

TPEEKSMPHWFDTWIRVERMSAIMPDQIAKAAKAKPVQKLDDDDDGDDTYKEERHNKYNS

LTRIKIPNPPKSFDDLKNIDTKKLLVRGLYRISFTTYKPGEVKGSFVASVGLLFPPGIPG

VSPLIHSNPEELQKQAIAAEESLKKAASDATK

>fig|174.55.peg.2321 LipL32 [Leptospira borgpetersenii FMAS_AP8]

MKKLSILAISAALFASITACGAFGGLPSLKSSFVLSESTVPGTNETVKTFLPYGSVINYY

GYVKPGQAPDGLVDGNKKAYYLYVWIPAVIAEMGVRMISPTGEIGEPGDGDLVSDAFKAA

TPEEKSMPHWFDTWIRVERMSAIMPDQIAKAAKAKPVQKLDDDDDGDDTYKEERHNKYNS

LTRIKIPNPPKSFDDLKNIDTKKLLVRGLYRISFTTYKPGEVKGSFVASVGLLFPPGIPG

VSPLIHSNPEELQKQAIAAEESLKKAASDATK

>fig|29507.8.peg.2904 LipL32 [Leptospira kirschneri FMAS_PN5]

MKKLSILAISVALFASITACGAFGGLPSLKSSFVLSEDTIPGTNETVKTLLPYGSVINYY

GYVKPGQAPDGLVDGNKKAYYLYVWIPAVIAEMGVRMISPTGEIGEPGDGDLVSDAFKAA

TPEEKSMPHWFDTWIRVERMSAIMPDQIAKAAKAKPVQKLDDDDDGDDTYKEERHNKYNS

LTRIKIPNPPKSFDDLKNIDTKKLLVRGLYRISFTTYKPGEVKGSFVASVGLLFPPGIPG

VSPLIHSNPEELQKQAIAAEESLKKAASDATK

>fig|173.274.peg.2174 LipL32 [Leptospira interrogans FMAS_PD1]

MKKLSILAISVALFASITACGAFGGLPSLKSSFVLSEDTIPGTNETVKTLLPYGSVINYY

GYVKPGQAPDGLVDGNKKAYYLYVWIPAVIAEMGVRMISPTGEIGEPGDGDLVSDAFKAA

TPEEKSMPHWFDTWIRVERMSAIMPDQIAKAAKAKPVQKLDDDDDGDDTYKEERHNKYNS

LTRIKIPNPPKSFDDLKNIDTKKLLVRGLYRISFTTYKPGEVKGSFVASVGLLFPPGIPG

VSPLIHSNPEELQKQAIAAEESLKKAASDATK

>fig|28184.24.peg.2384 LipL32 [Leptospira weilii FMAS_PD2]

MKKLSILVVSVALFASITACGAFGGLPSLKSSFVLSESTVPGTNETVKTFLPYGTVINYY

GYIKPGQAPDGLVDGSKKAYYLYVWVPAVIAEMGVRMISPTGEIGEPGDGDLVSDAFKAA

TPEEKSMPNWFDTWIRVERMSAIMPDQIAKAAKAKPVQKLDDDDDGDDTYKEERHSKYNS

LTRITIPNPPKSFDELKNIDTKKLLVRGLYRISFTTYKPGEVKGSFVASVGLLFPPGIPG

VSPLIHSNPEELQKQAIAAEESLKKAAASATK

**Nucleotide sequences LipL32**

>fig|174.58.peg.2326 LipL32 [Leptospira borgpetersenii FMAS_AP3]

atgaaaaaactttcgattttggcgatctccgctgcactctttgcaagcattaccgcttgt

ggtgcgttcggtggtttgccaagccttaaaagctcttttgttctgagcgagagcacagtt

ccaggaacaaacgaaaccgtaaaaacgttccttccctatggatctgtgatcaactattac

ggatatgtaaagccaggacaagcgccggacggtttagtcgacggaaacaaaaaagcatac

tatctctatgtttggatcccagctgtaatcgctgaaatgggagttcgtatgatttcccca

acaggcgaaatcggggaaccaggcgacggagacttagtaagcgatgctttcaaagcggca

accccggaagaaaaatcaatgccacattggtttgatacctggatccgtgtagaaagaatg

tcggcgattatgcctgaccaaatcgccaaagctgcgaaagcaaaacccgttcaaaaattg

gacgatgatgatgatggtgatgatacttataaagaagagagacacaataaatacaactct

cttactagaatcaagatcccaaatcctccaaaatcttttgacgatctgaaaaacatcgat

actaaaaaacttttagtaagaggtctttacagaatttctttcactacctacaaaccaggt

gaagtgaaaggatctttcgttgcatctgttggtctgctttttccaccaggtattccaggt

gtgagcccgttgatccactcaaatcctgaagaactgcaaaaacaagctatcgccgccgaa

gaatctttgaaaaaagctgcttccgacgcgactaagtaa

>fig|174.55.peg.2321 LipL32 [Leptospira borgpetersenii FMAS_AP8]

atgaaaaaactttcgattttggcgatctccgctgcactctttgcaagcattaccgcttgt

ggtgcgttcggtggtttgccaagccttaaaagctcttttgttctgagcgagagcacagtt

ccaggaacaaacgaaaccgtaaaaacgttccttccctatggatctgtgatcaactattac

ggatatgtaaagccaggacaagcgccggacggtttagtcgacggaaacaaaaaagcatac

tatctctatgtttggatcccagctgtaatcgctgaaatgggagttcgtatgatttcccca

acaggcgaaatcggggaaccaggcgacggagacttagtaagcgatgctttcaaagcggca

accccggaagaaaaatcaatgccacattggtttgatacctggatccgtgtagaaagaatg

tcggcgattatgcctgaccaaatcgccaaagctgcgaaagcaaaacccgttcaaaaattg

gacgatgatgatgatggtgatgatacttataaagaagagagacacaataaatacaactct

cttactagaatcaagatcccaaatcctccaaaatcttttgacgatctgaaaaacatcgat

actaaaaaacttttagtaagaggtctttacagaatttctttcactacctacaaaccaggt

gaagtgaaaggatctttcgttgcatctgttggtctgctttttccaccaggtattccaggt

gtgagcccgttgatccactcaaatcctgaagaactgcaaaaacaagctatcgccgccgaa

gaatctttgaaaaaagctgcttccgacgcgactaagtaa

>fig|29507.8.peg.2904 LipL32 [Leptospira kirschneri FMAS_PN5]

atgaaaaaactttcgattttggctatctccgttgcactctttgcaagcattaccgcttgt

ggtgctttcggtggtctgccaagcctaaaaagctcttttgttctgagcgaggacacaatc

ccagggacaaacgaaaccgtaaaaacgttacttccctacggatctgtgatcaactattac

ggatacgtaaagccaggacaagcgccggacggtttagtcgatggaaacaaaaaagcatac

tatctctatgtttggattcctgccgtaatcgctgaaatgggagttcgtatgatttcccca

acaggcgaaatcggtgaaccaggcgatggagacttagtaagcgacgctttcaaagcggct

accccagaagaaaaatcaatgccacattggtttgatacttggatccgtgtagaaagaatg

tcggcgattatgcctgaccaaatcgccaaagctgcgaaagcaaaacccgttcaaaagttg

gacgatgatgatgatggtgacgatacttataaagaagagagacacaacaagtacaactct

cttactagaatcaagatccctaatcctccaaaatcttttgacgatctgaaaaacatcgat

actaaaaaacttttagtaagaggtctttacagaatttctttcactacctacaaaccaggt

gaagtgaaaggatctttcgttgcatctgttggtctgcttttcccaccaggtattccaggt

gtgagcccgctgatccactcaaatcctgaagaattgcaaaaacaagctatcgctgctgaa

gagtctttgaaaaaagctgcttctgacgcgactaagtaa

>fig|173.274.peg.2174 LipL32 [Leptospira interrogans FMAS_PD1]

atgaaaaaactttcgattttggctatctccgttgcactctttgcaagcattaccgcttgt

ggtgctttcggtggtctgccaagcctaaaaagctcttttgttctgagcgaggacacaatc

ccagggacaaacgaaaccgtaaaaacgttacttccctacggatctgtgatcaactattac

ggatacgtaaagccaggacaagcgccggacggtttagtcgatggaaacaaaaaagcatac

tatctctatgtttggattcctgccgtaatcgctgaaatgggagttcgtatgatttcccca

acaggcgaaatcggtgagccaggcgacggagacttagtaagcgacgctttcaaagcggct

accccagaagaaaaatcaatgccacattggtttgatacttggatccgtgtagaaagaatg

tcggcgattatgcctgaccaaatcgccaaagctgcgaaagcaaaaccagttcaaaaattg

gacgatgatgatgatggtgacgatacttataaagaagagagacacaacaagtacaactct

cttactagaatcaagatccctaatcctccaaaatcttttgacgatctgaaaaacatcgac

actaaaaaacttttagtaagaggtctttacagaatttctttcactacctacaaaccaggt

gaagtgaaaggatctttcgttgcatctgttggtctgcttttcccaccaggtattccaggt

gtgagcccgctgatccactcaaatcctgaagaattgcaaaaacaagctatcgctgctgaa

gagtctttgaaaaaagctgcttctgacgcgactaagtaa

>fig|28184.24.peg.2384 LipL32 [Leptospira weilii FMAS_PD2]

atgaaaaaactttcgattttggtcgtctccgtcgcactctttgcaagcattaccgcttgt

ggtgcgttcggtggtctgccaagccttaaaagctcttttgtcctgagcgagagcacagtt

ccaggaacaaacgaaactgtaaaaacatttcttccttacggaacagtaatcaactactat

ggttacatcaaaccaggacaagcgccggacggtttagtcgatggaagcaaaaaagcatac

tacctctacgtttgggtacctgctgtgatcgctgaaatgggagttcgtatgatttcccca

acaggcgaaatcggtgaaccgggcgacggagatttagtaagcgacgctttcaaagcggca

accccagaagaaaaatcaatgcctaactggtttgatacctggattcgtgttgaaagaatg

tcagcgattatgcctgaccaaattgcaaaagctgcaaaggcaaaaccggttcaaaaactc

gatgacgatgatgacggagatgatacttataaagaagagagacattctaaatacaactct

cttacaagaatcaccatccctaatcctccgaaatcttttgacgaactgaaaaacatcgat

actaaaaaacttttagtaagaggtctttacagaatttctttcactacctacaaaccaggt

gaagtgaaaggatctttcgttgcatctgttggtctgcttttcccaccaggtattccaggt

gtgagtccgttgattcactcaaatcctgaagaactgcaaaaacaagccatagcagctgaa

gaatctttgaaaaaagcagcggcaagcgcgactaagtaa

**LigB amino acid sequences**

>fig|174.58.peg.3831 surface protein Lk90-like protein [Leptospira borgpetersenii FMAS_AP3]

MAEKINKSKDKKTRFFFKLTSILFLTFSLFFLETCAAWPIFSGAPGLLAGKKGGANNSFW

MLFLGVNDLLESDQAEIELDRIEISSPSSSLARGTSVYLNAIAIYKNNTHRDISSEGVWS

STDSSILKLLTLSQFKGMNLGSGNVNVAFQGKIASSTLTVTSSVLSNLIVTCVNQGNILP

VGIDRQCKLEGIFSDGNTQVLTSDPDTSWNVTQSSIVGVNSTGLVSGISPGSASITGSYH

GITSSLTITVSSATLSSIAVTPVNASYALGKVQQYTAIGTYSNQSTQDLTNQVSWASLNT

TVATVDNSAFSKGFLTTQSPGSASITATLGAITGQTQVNVTSAVLTSITITPANPSVAKG

RTLFSTATGIFSDGTVSDITNQVTWSSSLASVATVDNSAGLAGRITGIGVGTTNITAGIG

GVDSTISLDVTNAVLVSIQVASDSSSIARGTSTFVQAVGVYSDGSSQNISDQVAWSSSNS

SILQIANLNAVPKREMQSPSSGGLGTARITTTLEAISSYTDISVTAATLTSLEVSPTNPS

VATGLTVPFTATGVYTDGSNQNLTSQVTWNSSSTNRATISNASGSEGIALGSSAGTTNIS

ATLGVINSSSTVLTVTNAVLNSITITPALPSIAGGRSLNLTATGTYSDGNTQDLTTSVAW

TSVDSSIASVDNAVGRQGQTTGVTQGTTQISASLGGVSATINFTVTVAVLDSIQVTLEDS

PIAKGTFTRAIATGVFSDGSNLNISDQVVWDSSQTNVIQLGILEAGPKKKLMNSPANGSS

TTGTSRITATLGGVSGFADLTVIAPNLASIQIDPTHPSVANGLTQDFTATGIYSDGSNQN

LSDSVTWASSIPSVATISNASGSNGKATMLQAGSTNISASLGPVTSDPSVLTVTNAILTS

ITIAPTSSFNIAKGLNQNFVATGHYTDGSSRDLTSQVTWISSNTSAVTISNASGTQGRMT

AVDTGSTNISASLGGTSSANTNVTVTGAVLNLIQVSPADISMAKGNTKAYTAIGVYSDST

TLDVTSQVTWISSNTSIATISNASGHEGFVSTVSTGTTTITATLGAISNSTSLTVTAAVL

VSISVGPTNSFVYMTQTKNFTATGTYSDGTMQDLTTQVTWTSSDTTLGTVSNAFGTEGKV

TGIAAGAVTITSILGSISGNTSLSVIFLDAIAPTVANVVALTPSTVRITYSENVNEVQAK

VAANYKLALTSSVSGSCSDNSNFTSTFSVITVSSVSGSGSVFVLTLGSSQTSNVPYTIIV

NKSGVQDLSTSPNNLGCANYGDFLGQEQIKIVSASCANSNSVILNFSKTPKSGNNVSGSV

ECTGSTECASRYKIIGASDLGTINSVKMLDGIVCNGATADSAKVCVVHNLVQTGAQYTII

AADSVDGDGFDNTSWGSIRNSLDTENLQSSPRDRASFLGCGTSPVNFADGPVSIDPNSST

FGYLMDFNSKIYSGPNNSGNGALRFAYDGSAPESVQFSFEKDTTAQDGDPTNVSLNTASS

RENLITIPPYVTLGHSGCTSNNGTLSLGCGPDNESGRGVFVTGVLSGVSYLFVAGARTIA

DGLGQYFFDYLYYSADPSSNMSFKYMDLGSITGALTAGTSSLTVLNNRVFAGFAKSSNDG

VGLFGGLNAPDFGFVTFNSADSGTGFCTPGSNCDAFDGTKGKRVRIDFLPYFGGPSTGLL

GINNNAHPNWGYYIGVDSMFVFKNRIYAANGGLHAVGHNGSIIRSTTADPTAACTGPDSC

SNWVEIGPRTNTKWHNSPTNNWFSLELSQFYNLIPGDKAFAQFAEFNNNLYATRTVCVQS

SQALGIRSVPGTVAGCTDGTTTNRRAQLWKCDPTVSGNTSECDAADWSVVGDDGTGITNM

GDTNNRTITMVMKNGSYLYIGYDNSNGIKIYRTNTANPGSSSASWSQIAGSGLTDATNVQ

QIYSAVSVPSGSINYIYVSAGKNGVPVRTYRQQN

>fig|173.280.peg.3183 surface protein Lk90-like protein [Leptospira interrogans FMAS_AP6]

MTKHINKLRDKKTRSFLQFTFVFFLTFNLFFLESCAAWPIFSGAPGLLAGKKGGANNSLW

MLFLGVNNPLESEPSETELDRIEISVPNSSFARGTTLHLNATAIYKDNTHRDVSSEGSWS

STDSSILKLLTQSQFKGMNLGSGNVNVSFQGKNATTTLTVTSAVLSDLTVTCVNQGSPLP

VGIDRQCKLEGIFSDGSTQVLTSDPSASWNITQSSIAGVNTTGLVSGLSPGSTSITSSYG

SKTSSLNVTVSAATLSSIAVTPANSSYPLGKVQQYTAIGTYSNQSTQDLTNQVSWASLNT

SVATIDNSASSKGLLTTQSTGSANITATLGGITGQTQVNVTSAVLTSITITPANPSVANG

RTLYLTATGVFSDGTVSDITSQVTWSSSLTSVATADNSAGLSGRISGVGVGSTNITAAIG

GIDITISLNVTNATLESIQVVSDSHSIARGTSTFVQAIGIYSDGSSQNISDQVAWNSSNS

SILQISNLNAVPKREIQSPSSGGLGTARITATLEAISAYTDISVNAATLVSIEVSPTNPS

VAAGLTIPFTATGVYTDGSNQNLTSQVTWNSSNTNRATISNANGTQGIALGSSAGTTNIS

ATLGAVTSSATTLTVTNAVLNSITITPSIPSIAVGRSLNLTATGTYSDGSNQDLTTSVAW

TSADSSIASVDNASGRQGQATGVTQGNTQISSTLGGVSSTISFTVNAAVLDSIQVTLEDS

PIAKGTFTRAIATGVFSDGSNLNISDQVVWDSSQTNVIQLGVLETGPKKKLMNSPANGNS

TTGTSRITATLGGVSGYADLTVIAPSLTSIQIDPTHPSVANGLTQNFTATGVYSDGSNQN

LTDSVTWASSNPAVATISNASGTNGKATTLQTGSTNISASLGAVTSDPSVLTITNATLTN

ITIAPTSSFNIAKGLNQDFVATGYYTDGSSRDLTTQVTWNSSNASVSTISNANGTQGRMA

AVDTGSTNISASLGGTSSPNTNVTVTSAVLNSIQISPADISVAKGNTKVYTAIGVYSDFS

TLDVTSQVTWTSSSVSVATISNASGHEGFATTVGTGTSTITATLGGISNSTSLTVTAAVL

VSLSVGPTNSFVYMTQTKNFTATGTYSDGTMQDLTTQVTWTSSDTTLGTISNSFGIEGRA

TGIAAGAVTLTATLGSISGNTSLTVIFLDTIPPTVTNVVALTPTTVRITYSENVNEIQAK

IAANYKLALTSSVTGSCSDNSNFTSTSSVITVSSVSGSGSVFVLTLGSSQTSNAPYTILV

NKLGVQDLSTTPNNLGCANYGDFLGQEQIKIVSASCANSNSVILNFSKAPKSGNNITGSA

ECTGSTECSNRYKISGASDLGTINSVKVLDGIVCNGATADSAKVCVVHNLVQTGAQYTII

TADSVDGDGFDNSSWGSIRNSLDTENLQSSPRDRASFLGCGTSPVNFADGPISIDPNSST

FGYLIDFNSKIYSGPNNSGNGALRFAYDGSIPESVQFSFEKDTTVQDGDATNVSSNLASS

RENSISVPPYVTLGHSGCTTNNGTLSLGCGPDNENGRGVFATGILSSASYLFVAGAKTIA

DGLGQYLFDYLYYSADPSSNMSYKYIDLGSITGTLTAGTSSLTVLNNRVFAGFAKSSNDG

IGLFGGLNAPDFGFVTFNSADSGTGFCTPGSNCDAFDGTKGKRIRIDFLPYFGGPSTGLL

GINNNAHPNWAYYIGVDSMFVFKNRIYAANGGLHSVGHNGSIIRSTTTDPTAACTGPDSC

SNWVEIGPRTNTKWHNSPTNNWFSLELNQFYNLIPGDKAFAQFAEFNNNLYVTRTICVQS

SQATGIRTSPGTVAGCTDGTTTNRRAQLWKCDPTISGNTSECDAADWSVVGDDGTGITNM

GDSTNRTITMVMKNGSYLYIGYDNPNGIRIYRTNVANPGSSSASWSQIAGNGLTDSTNVQ

QIYSAVSVPSGSINYIYVSVGKSNVPVRTYRQQN

>fig|29507.8.peg.3299 surface protein Lk90-like protein [Leptospira kirschneri FMAS_PN5]

MTKHINKLRDKKTRSFLQFTFVFFLTFNLFFLESCAAWPIFSGAPGLLAGKKGGANNSLW

MLFLGVNNPLESEPSETELDRIEISVPNSSFARGTTLHLNATAIYKDNTHRDVSSEGSWS

STDSSILKLLTQSQFKGMNLGSGNVNVSFQGKNATTTLTVTSAVLSDLTVTCVNQGSPLP

VGIDRQCKLEGIFSDGSTQVLTSDPSASWNIIQSSIAGVNTTGLVSGLSPGSTSITSSYG

SKTSSLNVTVSAATLSSIAVTPANSSYPLGKVQQYTAIGTYSNQSTQDLTNQVSWASLNT

SVATIDNSASSKGLLTTQSTGSANITATLGGITGQTQVNVTSAVLTSITITPANPSIANG

RTLYLTATGVFSDGTVSDITSQVTWSSSLTSVATADNSAGLSGRISGVGVGSTNITAAIG

GIDITISLNVTNATLESIQVVSDSHSIARGTSTFVQAIGVYSDGSSQNISDQVAWNSSNS

LILQISNLNAVPKREIQSPSSGGLGTARITATLEAISAYTDISVNAATLVSIEVSPTNPS

VAAGLTIPFTATGVYTDGSNQNLTSQVTWNSSNTNRATISNANGTQGIALGSSAGTTNIS

ATLGAVTSSATTLTVTNAVLNSITITPSIPSIVVGRSLNLTATGTYSDGSNQDLTTSVAW

TSADSSIASVDNASGRQGQATGVTQGNTQISSTLGGVSSTISFTVNAAVLDSIQVTLEDS

PIAKGTFTRAIATGVFSDGSNLNISDQVVWDSSQTNVIQLGVLETGPKKKLMNSPANGNS

TTGTSRITATLGGVSGYADLTVIAPSLTSIQIDPTHPSVANGLTQNFTATGVYSDGSNQN

LTDSVTWASSNPAVATISNASGTNGKATTLQTGSTNISASLGAVTSDPSVLTITNATLTN

ITIAPTSSFNIAKGLNQDFVATGYYTDGSSRDLTTQVTWNSSNASVSTISNANGTQGRMA

AVDTGSTNISASLGGTSSPNTNVTVTSAVLNSIQISPADISVAKGNTKVYTAIGVYSDFS

TLDVTSQVTWTSSSVSVATISNASGHEGFATTVGTGTSTITATLGGISNSTSLTVTAAVL

VSLSVGPTNSFVYMTQTKNFTATGTYSDGTMQDLTTQVTWTSSDTTLGTISNSFGIEGRA

TGIAAGAVTLTATLGSISGNTSLTVIFLDTIPPTVTNVVALTPTTVRITYSENVNEIQAK

IAANYKLALTSSVTGSCSDNSNFTSTSSVITVSSVSGSGSVFVLTLGSSQTSNAPYTILV

NKLGVQDLSTTPNNLGCANYGDFLGQEQIKIVSASCANSNSVILNFSKAPKSGNNITGSA

ECTGSTECSNRYKISGASDLGTINSVKVLDGIVCNGATADSAKVCVVHNLVQTGAQYTII

TADSVDGDGFDNSSWGSIRNSLDTENLQSSPRDRASFLGCGTSPVNFADGPISIDPNSST

FGYLIDFNSKIYSGPNNSGNGALRFAYDGSIPESVQFSFEKDTTVQDGDATNVSSNLASS

RENSISVPPYVTLGHSGCTTNNGTLSLGCGPDNENGRGVFATGILSSASYLFVAGAKTIA

DGLGQYLFDYLYYSADPSSNMSYKYIDLGSITGTLTAGTSSLTVLNNRVFAGFAKSSNDG

IGLFGGLNAPDFGFVTFNSADSGTGFCTPGSNCDAFDGTKGKRIRIDFLPYFGGPSTGLL

GINNNAHPNWAYYIGVDSMFVFKNRIYAANGGLHSVGHNGSIIRSTTTDPTAACTGPDSC

SNWVEIGPRTNTKWHNSPTNNWFSLELNQFYNLIPGDKAFAQFAEFNNNLYVTRTICVQS

SQATGIRTSPGTVAGCTDGTTTNRRAQLWKCDPTISGNTSECDAADWSVVGDDGSGITNM

GDSTNRTITMVMKNGSYLYIGYDNPNGIRIYRTNVANPGSSSASWSQIAGNGLTDSTNVQ

QIYSAVSVPSGSINYIYVSVGKSNVPVRTYRQQN

>fig|28184.25.peg.365 surface protein Lk90-like protein [Leptospira weilii FMAS_RT1]

MLVFEMGSTQSSGSANVTATLGAITGQTQVNVTSAVLTSITITPANPSVANGRTLFLTAT

GIFSDGTASDITNQVTWSSSLASVATADNSAGLSGRITGIGVGTTNITAGIGGVDNTLSL

SVTNAVLESVQVVSDSSSIARGTSTFVQAIGVYSDGSSQNISDQVAWSSSNSSVLQIANL

NAIPKREVQSPSSGGFGTARITATLEAISSHTDISVTAATLISLEVSPTNPSVAAGLTVP

FTATGVYTDGSNQNLTSQATWNSSNTNRATISNASGSEGIALGSSAGTTNISATLGAITS

SSTTLTVTNAVLNSITITPALPSIAGGRSLNLTATGAYSDGNTQDLTTSVAWTSADSSIA

SVDNAAGRQGQTTGVAQGTTQISALLGGVSATINFTVTAAVLDSIQVTLEDSPIAKGTFT

RAIATGVFSDGSNLNISDQVVWDSSQTNVIQLGILEAGPKKKLMNSPANGSSTTGTSRIT

ATLGGVSGFADLTVIAPNLVSIQIDPTHPNVANGLSQNFTATGVYSDGSNQNLTDSVTWA

SSNPAIATISNASGSNGKATMLQTGSTNISASLGPVTSDPSVLTVTSATLTSITIAPTSS

FNIAKGLNQNFVATGYYTDGSSRDLTSQVTWTSSNTSTAMISNASGTQGKMTAVDTGSTN

ISASLGGTSSANTNVTVTAAVLNSIQVSPADISVAKGNTKAYAAIGVYSDSTTLDVTSQV

TWTSSNTSIATISNASGHEGLATTVSAGTTTITATLGAVSNSTSLTVTAAVLVSLSVGPT

NSFVYMTQTKYFTATGTYSDGTMQDLTTQVTWTSSDTTLGTVSNAFGTEGKATGIAAGAV

TITATLGSISGNTSLSVIFLDTVAPTVTNVVALTPTTVRITYSENVNEIQAKTAANYKLA

LTSAVSGSCSDNSNFTSTSSVITVSSVSGSGSVFVLTLGSSQTSNAPYTILVNKSGVQDL

STSPNNLGCANYGDFLGQEQIKIVSASCANSSSVILNFSKAPKSGNNISGSAECTGSTEC

ASRYKIAGASDLGTINSAKTLDGIVCNGATADSAKVCVVHNLVQTGAQYTIIAADSADGD

GFDNASWGSIRNSLDTENLQSSPRDRASFLGCGTSPVNFADGPISIDPNSSTFGYLMDFN

SKIYSGPNNSGNGALRFAYDGSAPESVQFSFEKDTTAQDGDPTNVSSNIASSRENSIAIP

PYVTLGHSGCTPNNGTLSLGCGPDNESGRGAFVTGILSSVSYLFVAGARTVADGLGQYFF

DYLYYSADPSSNMSFKYIDLGSITGTLTAGTSSLTVLNNRVFAGFAKSSNDGVGLFGGLN

APDFGFVTFNSADSGTGFCTPGSNCDAFDGTKGKRIRIDFLPYFGGPSTGLLGLNNNAHP

NWGYYIGVDSMFVFKNRIYAANGGLHAVGHNGSIIRSTTADPTAACTGPDSCSNWVEIGP

RTNTKWHNSPTNNWFSLELNQFYNLIPGDKAFAQFAEFNNNLYVTRTVCVQSSQAIGIRT

SAGTVAGCTDGTTTNRRAQLWKCDPTISGNTSECDAADWSVVGDDGTGITNMGDSTNRTI

TMVMKNGSYLYVGYDNSNGIRIYRTNVANPGSSSASWSQIAGNGLTDATNVQQIYSAVSV

PSGSINYIYVSAGKSGVPVRTYRQQN

>fig|174.59.peg.3933 surface protein Lk90-like protein [Leptospira borgpetersenii FMAS_AP4]

MAEKINKSKDKKTRFFFKLTSILFLTFSLFFLETCAAWPIFSGAPGLLAGKKGGANNSFW

MLFLGVNDLLESDQAEIELDRIEISSPSSSLARGTSVYLNAIAIYKNNTHRDISSEGVWS

STDSSILKLLTLSQFKGMNLGSGNVNVAFQGKIASSTLTVTSSVLSNLIVTCVNQGNILP

VGIDRQCKLEGIFSDGNTQVLTSDPDTSWNVTQSSIVGVNSTGLVSGISPGSASITGSYH

GITSSLTITVSSATLSSIAVTPVNASYALGKVQQYTAIGTYSNQSTQDLTNQVSWASLNT

TVATVDNSAFSKGFLTTQSPGSASITATLGAITGQTQVNVTSAVLTSITITPANPSVAKG

RTLFSTATGIFSDGTVSDITNQVTWSSSLASVATVDNSAGLAGRITGIGVGTTNITAGIG

GVDSTISLDVTNAVLVSIQVASDSSSIARGTSTFVQAVGVYSDGSSQNISDQVAWSSSNS

SILQIANLNAVPKREMQSPSSGGLGTARITTTLEAISSYTDISVTAATLTSLEVSPTNPS

VATGLTVPFTATGVYTDGSNQNLTSQVTWNSSSTNRATISNASGSEGIALGSSAGTTNIS

ATLGVINSSSTVLTVTNAVLNSITITPALPSIAGGRSLNLTATGTYSDGNTQDLTTSVAW

TSVDSSIASVDNAVGRQGQTTGVTQGTTQISASLGGVSATINFTVTVAVLDSIQVTLEDS

PIAKGTFTRAIATGVFSDGSNLNISDQVVWDSSQTNVIQLGILEAGPKKKLMNSPANGSS

TTGTSRITATLGGVSGFADLTVIAPNLASIQIDPTHPSVANGLTQDFTATGIYSDGSNQN

LSDSVTWASSIPSVATISNASGSNGKATMLQAGSTNISASLGPVTSDPSVLTVTNAILTS

ITIAPTSSFNIAKGLNQNFVATGHYTDGSSRDLTSQVTWISSNTSIATISNASGHEGFVS

TVSTGTTTITATLGAISNSTSLTVTAAVLVSISVGPTNSFVYMTQTKNFTATGTYSDGTM

QDLTTQVTWTSSDTTLGTVSNAFGTEGKVTGIAAGAVTITSILGSISGNTSLSVIFLDAI

APTVANVVALTPSTVRITYSENVNEVQAKVAANYKLALTSSVSGSCSDNSNFTSTFSVIT

VSSVSGSGSVFVLTLGSSQTSNVPYTIIVNKSGVQDLSTSPNNLGCANYGDFLGQEQIKI

VSASCANSNSVILNFSKTPKSGNNVSGSVECTGSTECASRYKIIGASDLGTINSVKMLDG

IVCNGATADSAKVCVVHNLVQTGAQYTIIAADSVDGDGFDNTSWGSIRNSLDTENLQSSP

RDRASFLGCGTSPVNFADGPVSIDPNSSTFGYLMDFNSKIYSGPNNSGNGALRFAYDGSA

PESVQFSFEKDTTAQDGDPTNVSLNTASSRENLITIPPYVTLGHSGCTSNNGTLSLGCGP

DNESGRGVFVTGVLSGVSYLFVAGARTIADGLGQYFFDYLYYSADPSSNMSFKYMDLGSI

TGALTAGTSSLTVLNNRVFAGFAKSSNDGVGLFGGLNAPDFGFVTFNSADSGTGFCTPGS

NCDAFDGTKGKRVRIDFLPYFGGPSTGLLGINNNAHPNWGYYIGVDSMFVFKNRIYAANG

GLHAVGHNGSIIRSTTADPTAACTGPDSCSNWVEIGPRTNTKWHNSPTNNWFSLELSQFY

NLIPGDKAFAQFAEFNNNLYATRTVCVQSSQALGIRSVPGTVAGCTDGTTTNRRAQLWKC

DPTVSGNTSECDAADWSVVGDDGTGITNMGDTNNRTITMVMKNGSYLYIGYDNSNGIKIY

RTNTANPGSSSASWSQIAGSGLTDATNVQQIYSAVSVPSGSINYIYVSAGKNGVPVRTYR

QQN

**Lig C nucleotide sequences**

>fig|174.58.peg.3831 surface protein Lk90-like protein [Leptospira borgpetersenii FMAS_AP3]

atggctgagaagattaacaaatccaaagataaaaaaacacgattcttctttaaacttacc

tcgattcttttcctcacattcagtttattctttttggaaacttgcgcggcttggccgatt

ttttcaggcgctcccggtttactggcgggtaaaaaaggaggagccaacaattctttttgg

atgctctttctaggagtaaacgatctgcttgaatcggatcaagccgaaattgaattggat

cgaatcgaaatttcatccccttcttcgagtttggctcggggaacctctgtgtatttaaat

gcgatcgctatctataaaaacaatactcatcgagatatttcttcggaaggagtttggtcc

tctacggattcaagcattctcaagttgttaactctatctcagttcaaagggatgaatctc

ggttccggaaacgtaaacgttgcgtttcaaggaaagatcgcttcttctacattaaccgtt

acatcttctgttttgtccaatctgatcgttacctgtgtcaatcagggaaatatcttaccg

gtgggaatcgatcgccaatgcaaattagaaggaattttttcagatggaaatacacaggtt

ttgacttccgatcccgatacgtcttggaacgtcactcaatcttccattgttggagtgaat

tccactggtcttgtttcaggaatttctccaggttccgcttcgattactggttcgtaccat

ggtatcacttccagtttgaccattacggtaagttccgcaaccttaagttcgatcgccgtg

actcctgtaaatgctagttatgctctcggtaaagtgcaacagtataccgcaattggaacc

tacagtaaccaatccactcaggatctgacaaatcaagtttcttgggcttctttaaataca

accgtcgctactgtggataactctgcgttctccaaaggttttctcactacacaatctcca

ggctctgcgagtatcacggccactttaggcgcgattactggtcaaacccaggttaacgtt

acttcggcggttcttacaagtattacgattactcctgcgaatccaagcgttgctaaagga

agaactttgttttcgaccgcaactggaattttttcggatggtacggtttccgatatcaca

aatcaagtaacttggtccagttctttggcgagtgtcgctactgtggataactccgcgggt

ttagccggtagaattacaggaatcggagttggtactacaaatatcaccgcggggatcgga

ggagtggacagtactatttctttagatgtcacgaacgctgttttagtatcgattcaggtg

gcttccgattcttcttcgattgcccgaggcacgtctacgttcgttcaggcggtcggagtt

tactcggacggttcttctcaaaacatcagcgatcaggtcgcttggagtagttctaattct

tccatcttgcaaatcgcgaacttgaatgcggttccgaaacgagaaatgcagtctccttcc

tctggaggtttgggtacggcaagaatcacgactactttggaagcgatttcctcgtatacg

gatatctcggtaactgcggcaactttaacttcgcttgaagtctctccgacaaatccttcg

gttgcgaccggtcttacggttcctttcactgctacaggagtttatacggacggtagcaat

cagaatttaacgtctcaagtgacttggaattcttctagcacaaatcgagccacgattagt

aatgcttcgggttctgaaggaattgccttaggttcttctgctgggactacgaatatttct

gccacgttaggcgttattaattcttcctctacggttcttactgtcacaaatgcggtttta

aattcgatcacgatcactccggctcttccttcgattgcaggtggaagaagtttgaatctt

accgcgacgggaacctattcggatggaaatactcaggatttaactacttctgttgcctgg

acgagtgtggattcttccatcgcttccgtagacaacgctgtgggtaggcagggacaaaca

accggtgtcacacagggaacaactcaaatcagcgcttcgttgggaggagtttccgctacg

atcaattttacggtgaccgtcgcagttttggattccattcaagtgaccttggaagattct

ccgattgcgaaaggtacatttaccagggcaatcgcgaccggtgttttttccgacggtagc

aatttgaatatcagtgatcaagttgtttgggatagttctcaaacaaatgtgatccagctt

gggattttagaagccggtcctaaaaagaaactgatgaattctcccgcaaacggaagcagt

accacggggacttcaaggatcactgcaactctcggtggggtgagcggatttgccgatctt

accgtaattgctccgaacttggccagcattcagatcgatcccactcatccgagcgttgcc

aacggtttgactcaggattttaccgctacgggtatttattcggacggtagcaatcagaat

ctatccgattccgtaacttgggcttcttctattccgtctgttgcgacgatcagtaacgct

tcgggatcgaacggtaaggcgacgatgcttcaagcgggatcgactaatatcagcgcgagt

ttgggcccggtgacttccgatccgagcgtccttacagttacaaacgcaattttaacgagc

atcacaatcgctccgacttcttctttcaacatcgcgaaggggttaaatcaaaactttgta

gcgacgggtcattatacggacggttcatccagagatctgacatctcaagtgacttggatt

tcttcgaatacttccgccgttacgattagcaacgcgagcggaactcaaggaagaatgacg

gcggtcgatacgggttctactaacatttccgcgtctttaggaggaacgtcgagtgcgaac

acgaacgtaacagtgacgggggcggttttgaatttgattcaggtttcgcctgctgatatc

agcatggcaaaaggaaacacgaaggcgtacactgcgatcggtgtgtattcggattctacg

acattagacgtcacgtctcaagtgacttggatttcttcgaatacttccattgctacgatc

agtaacgcgagcggtcacgaaggtttcgtttctaccgtttctaccggaacgaccacgatt

accgcgactctgggagcgatttccaattctacaagcctaaccgttacagccgccgtactt

gtttctatttcagtaggtcctacgaacagctttgtttatatgacacaaacgaaaaatttt

acggctacgggaacgtactccgatggaacgatgcaggacctgacgacccaagtgacttgg

acttcttccgacacaactctgggaacggtgagcaacgctttcggaacggaaggtaaggtc

acgggaattgcggctggagccgtgacgatcacttcaattctcggaagcattagcggaaat

acttctttgtccgtgatctttttggatgcgattgctccgacggtcgcgaacgtagtggct

ttgactccttctacggtgaggatcacatattcggaaaacgtaaacgaagttcaggctaaa

gttgcggccaattacaaattggctcttacgtcctctgtgagcggaagttgttcggacaac

agtaactttacttctactttttccgtgattaccgtctcttctgtgagcggaagcgggtct

gtattcgttttaacactcgggtcttctcaaacgtctaatgttccttatacgattatagtg

aataagtcgggggtacaggatctttccactagtccgaacaatttgggttgtgcgaactat

ggtgactttttaggacaggagcagatcaaaatcgtttccgcttcctgtgcgaattccaac

tcggtaatcctgaatttctccaaaacccctaagtccggaaataacgtttcaggttctgtg

gaatgtaccggttctacggaatgtgcgagtcgttacaaaattatcggcgcaagcgatctg

ggaacgatcaatagcgttaagatgttggatggaattgtttgtaacggagcgacagccgat

tccgcaaaagtctgcgtcgtacacaatctagtgcaaacgggcgcacaatatactatcatt

gccgcagattctgtagacggagatggatttgacaacacaagttggggatcaattcgaaat

tctttggatacggagaatcttcaatcttctccgagagatagagcttccttcttaggatgt

ggaacttctcccgtaaactttgcggatggaccggtttccatcgatccgaactcgtccacg

ttcggatatctcatggatttcaattctaaaatttattcggggccgaacaattccggaaac

ggagcactcaggttcgcttatgacggaagcgctcccgaatcggttcaattctcctttgaa

aaggatacgaccgcccaggatggtgatccgacgaatgtaagtttgaataccgcttcttct

cgggagaatttgatcacgattccgccttacgtaacattaggacattccggatgtacctca

aacaacggaaccctttctcttggatgcggtccggataacgaaagtggaagaggagtgttc

gttacgggagttctttccggtgtatcttatctttttgtcgcaggagcgagaaccatagcg

gatggactcggacaatactttttcgattatctgtattattccgcagatccttcttccaac

atgagtttcaaatacatggatctcggctcgatcacgggggctttgaccgctggaacttcc

tcgttgacggttctcaacaatagagtgtttgccggttttgcgaaatctagtaacgacggg

gtcggattgttcgggggactaaacgcacctgatttcggattcgtaaccttcaactctgcg

gattccggaaccggattctgtactcccggttccaactgcgatgcgtttgacggaaccaaa

ggcaaaagagtccggatcgatttccttccttacttcggaggaccgtcaacgggtttatta

ggaatcaataataacgcacatccgaattgggggtattatatcggagtcgattctatgttc

gtatttaaaaatcgtatctacgctgcaaacgggggattacacgcggtagggcataacgga

tctatcatacgttctaccacggccgatcctacggcggcttgtacaggacccgattcttgt

tcgaactgggtggagattggaccaagaaccaatacgaaatggcataatagtccgacgaac

aattggttctctttagagttgagtcaattttacaacttgattcccggggacaaagctttt

gcacaattcgccgagttcaacaacaacctttatgcaacgagaactgtctgcgttcaaagt

tcgcaagcgctcggaatccgaagcgtgccgggaaccgtcgcgggttgtacggatggaacg

accacgaaccgaagagcgcaactctggaaatgcgatccgaccgtatcgggaaacacgagc

gaatgcgacgcagcggattggtccgtggtaggcgacgacggaacgggaatcacgaatatg

ggagatacgaacaaccgaacgatcacgatggtgatgaaaaacggatcgtatctctacatc

ggatacgacaattcgaacggaatcaaaatctacagaacgaacacggccaatcccggatct

tcgtcagcgtcttggagccagatcgcgggcagcggactgacggatgcgacaaacgtccaa

cagatctattcggcggtctcggttccatcgggaagcatcaactacatctacgtcagcgct

gggaaaaacggagttccggttcggacgtatcgtcaacagaattga

>fig|174.53.peg.3831 surface protein Lk90-like protein [Leptospira borgpetersenii FMAS_AP5]

atggctgagaagattaacaaatccaaagataaaaaaacacgattcttctttaaacttacc

tcgattcttttcctcacattcagtttattctttttggaaacttgcgcggcttggccgatt

ttttcaggcgctcccggtttactggcgggtaaaaaaggaggagccaacaattctttttgg

atgctctttctaggagtaaacgatctgcttgaatcggatcaagccgaaattgaattggat

cgaatcgaaatttcatccccttcttcgagtttggctcggggaacctctgtgtatttaaat

gcgatcgctatctataaaaacaatactcatcgagatatttcttcggaaggagtttggtcc

tctacggattcaagcattctcaagttgttaactctatctcagttcaaagggatgaatctc

ggttccggaaacgtaaacgttgcgtttcaaggaaagatcgcttcttctacattaaccgtt

acatcttctgttttgtccaatctgatcgttacctgtgtcaatcagggaaatatcttaccg

gtgggaatcgatcgccaatgcaaattagaaggaattttttcagatggaaatacacaggtt

ttgacttccgatcccgatacgtcttggaacgtcactcaatcttccattgttggagtgaat

tccactggtcttgtttcaggaatttctccaggttccgcttcgattactggttcgtaccat

ggtatcacttccagtttgaccattacggtaagttccgcaaccttaagttcgatcgccgtg

actcctgtaaatgctagttatgctctcggtaaagtgcaacagtataccgcaattggaacc

tacagtaaccaatccactcaggatctgacaaatcaagtttcttgggcttctttaaataca

accgtcgctactgtggataactctgcgttctccaaaggttttctcactacacaatctcca

ggctctgcgagtatcacggccactttaggcgcgattactggtcaaacccaggttaacgtt

acttcggcggttcttacaagtattacgattactcctgcgaatccaagcgttgctaaagga

agaactttgttttcgaccgcaactggaattttttcggatggtacggtttccgatatcaca

aatcaagtaacttggtccagttctttggcgagtgtcgctactgtggataactccgcgggt

ttagccggtagaattacaggaatcggagttggtactacaaatatcaccgcggggatcgga

ggagtggacagtactatttctttagatgtcacgaacgctgttttagtatcgattcaggtg

gcttccgattcttcttcgattgcccgaggcacgtctacgttcgttcaggcggtcggagtt

tactcggacggttcttctcaaaacatcagcgatcaggtcgcttggagtagttctaattct

tccatcttgcaaatcgcgaacttgaatgcggttccgaaacgagaaatgcagtctccttcc

tctggaggtttgggtacggcaagaatcacgactactttggaagcgatttcctcgtatacg

gatatctcggtaactgcggcaactttaacttcgcttgaagtctctccgacaaatccttcg

gttgcgaccggtcttacggttcctttcactgctacaggagtttatacggacggtagcaat

cagaatttaacgtctcaagtgacttggaattcttctagcacaaatcgagccacgattagt

aatgcttcgggttctgaaggaattgccttaggttcttctgctgggactacgaatatttct

gccacgttaggcgttattaattcttcctctacggttcttactgtcacaaatgcggtttta

aattcgatcacgatcactccggctcttccttcgattgcaggtggaagaagtttgaatctt

accgcgacgggaacctattcggatggaaatactcaggatttaactacttctgttgcctgg

acgagtgtggattcttccatcgcttccgtagacaacgctgtgggtaggcagggacaaaca

accggtgtcacacagggaacaactcaaatcagcgcttcgttgggaggagtttccgctacg

atcaattttacggtgaccgtcgcagttttggattccattcaagtgaccttggaagattct

ccgattgcgaaaggtacatttaccagggcaatcgcgaccggtgttttttccgacggtagc

aatttgaatatcagtgatcaagttgtttgggatagttctcaaacaaatgtgatccagctt

gggattttagaagccggtcctaaaaagaaactgatgaattctcccgcaaacggaagcagt

accacggggacttcaaggatcactgcaactctcggtggggtgagcggatttgccgatctt

accgtaattgctccgaacttggccagcattcagatcgatcccactcatccgagcgttgcc

aacggtttgactcaggattttaccgctacgggtatttattcggacggtagcaatcagaat

ctatccgattccgtaacttgggcttcttctattccgtctgttgcgacgatcagtaacgct

tcgggatcgaacggtaaggcgacgatgcttcaagcgggatcgactaatatcagcgcgagt

ttgggcccggtgacttccgatccgagcgtccttacagttacaaacgcaattttaacgagc

atcacaatcgctccgacttcttctttcaacatcgcgaaggggttaaatcaaaactttgta

gcgacgggtcattatacggacggttcatccagagatctgacatctcaagtgacttggatt

tcttcgaatacttccgccgttacgattagcaacgcgagcggaactcaaggaagaatgacg

gcggtcgatacgggttctactaacatttccgcgtctttaggaggaacgtcgagtgcgaac

acgaacgtaacagtgacgggggcggttttgaatttgattcaggtttcgcctgctgatatc

agcatggcaaaaggaaacacgaaggcgtacactgcgatcggtgtgtattcggattctacg

acattagacgtcacgtctcaagtgacttggatttcttcgaatacttccattgctacgatc

agtaacgcgagcggtcacgaaggtttcgtttctaccgtttctaccggaacgaccacgatt

accgcgactctgggagcgatttccaattctacaagcctaaccgttacagccgccgtactt

gtttctatttcagtaggtcctacgaacagctttgtttatatgacacaaacgaaaaatttt

acggctacgggaacgtactccgatggaacgatgcaggacctgacgacccaagtgacttgg

acttcttccgacacaactctgggaacggtgagcaacgctttcggaacggaaggtaaggtc

acgggaattgcggctggagccgtgacgatcacttcaattctcggaagcattagcggaaat

acttctttgtccgtgatctttttggatgcgattgctccgacggtcgcgaacgtagtggct

ttgactccttctacggtgaggatcacatattcggaaaacgtaaacgaagttcaggctaaa

gttgcggccaattacaaattggctcttacgtcctctgtgagcggaagttgttcggacaac

agtaactttacttctactttttccgtgattaccgtctcttctgtgagcggaagcgggtct

gtattcgttttaacactcgggtcttctcaaacgtctaatgttccttatacgattatagtg

aataagtcgggggtacaggatctttccactagtccgaacaatttgggttgtgcgaactat

ggtgactttttaggacaggagcagatcaaaatcgtttccgcttcctgtgcgaattccaac

tcggtaatcctgaatttctccaaaacccctaagtccggaaataacgtttcaggttctgtg

gaatgtaccggttctacggaatgtgcgagtcgttacaaaattatcggcgcaagcgatctg

ggaacgatcaatagcgttaagatgttggatggaattgtttgtaacggagcgacagccgat

tccgcaaaagtctgcgtcgtacacaatctagtgcaaacgggcgcacaatatactatcatt

gccgcagattctgtagacggagatggatttgacaacacaagttggggatcaattcgaaat

tctttggatacggagaatcttcaatcttctccgagagatagagcttccttcttaggatgt

ggaacttctcccgtaaactttgcggatggaccggtttccatcgatccgaactcgtccacg

ttcggatatctcatggatttcaattctaaaatttattcggggccgaacaattccggaaac

ggagcactcaggttcgcttatgacggaagcgctcccgaatcggttcaattctcctttgaa

aaggatacgaccgcccaggatggtgatccgacgaatgtaagtttgaataccgcttcttct

cgggagaatttgatcacgattccgccttacgtaacattaggacattccggatgtacctca

aacaacggaaccctttctcttggatgcggtccggataacgaaagtggaagaggagtgttc

gttacgggagttctttccggtgtatcttatctttttgtcgcaggagcgagaaccatagcg

gatggactcggacaatactttttcgattatctgtattattccgcagatccttcttccaac

atgagtttcaaatacatggatctcggctcgatcacgggggctttgaccgctggaacttcc

tcgttgacggttctcaacaatagagtgtttgccggttttgcgaaatctagtaacgacggg

gtcggattgttcgggggactaaacgcacctgatttcggattcgtaaccttcaactctgcg

gattccggaaccggattctgtactcccggttccaactgcgatgcgtttgacggaaccaaa

ggcaaaagagtccggatcgatttccttccttacttcggaggaccgtcaacgggtttatta

ggaatcaataataacgcacatccgaattgggggtattatatcggagtcgattctatgttc

gtatttaaaaatcgtatctacgctgcaaacgggggattacacgcggtagggcataacgga

tctatcatacgttctaccacggccgatcctacggcggcttgtacaggacccgattcttgt

tcgaactgggtggagattggaccaagaaccaatacgaaatggcataatagtccgacgaac

aattggttctctttagagttgagtcaattttacaacttgattcccggggacaaagctttt

gcacaattcgccgagttcaacaacaacctttatgcaacgagaactgtctgcgttcaaagt

tcgcaagcgctcggaatccgaagcgtgccgggaaccgtcgcgggttgtacggatggaacg

accacgaaccgaagagcgcaactctggaaatgcgatccgaccgtatcgggaaacacgagc

gaatgcgacgcagcggattggtccgtggtaggcgacgacggaacgggaatcacgaatatg

ggagatacgaacaaccgaacgatcacgatggtgatgaaaaacggatcgtatctctacatc

ggatacgacaattcgaacggaatcaaaatctacagaacgaacacggccaatcccggatct

tcgtcagcgtcttggagccagatcgcgggcagcggactgacggatgcgacaaacgtccaa

cagatctattcggcggtctcggttccatcgggaagcatcaactacatctacgtcagcgct

gggaaaaacggagttccggttcggacgtatcgtcaacagaattga

>fig|173.275.peg.2433 surface protein Lk90-like protein [Leptospira interrogans FMAS_KG1]

atgactaagcatatcaacaaactcagagataaaaaaacgcggtcttttcttcagtttact

tttgttttttttctaacattcaatttattttttttggaaagttgcgcggcttggccgatt

ttttcaggcgctccgggtttgttagcaggtaaaaaaggtggggcaaacaattcactttgg

atgctttttttaggagtaaacaacccacttgaatcggaaccatccgagacagagttagat

cggatcgaaatttccgtaccaaattcaagttttgctcgaggtaccactttacatctaaac

gccaccgccatctacaaagacaatactcatcgagatgtttcctcggaaggatcctggtcc

tctacggattcgagcattctcaagctattaacacaatcccaattcaaaggaatgaatcta

ggttctggaaatgtaaacgtatcctttcaaggaaaaaatgcaaccacaacgttaaccgtc

acatctgctgttttgtccgatttaaccgtaacttgtgtaaatcaaggtagtccattacct

gttggaatcgatcgtcaatgtaaattggaaggaattttttcagacggtagcactcaggtt

ttaacttccgatccaagtgcgtcttggaacataactcaatcttctatcgcaggtgtaaac

acatcaggtttagtttccgggctttctcctggcagcacttctattactagttcctatgga

agcaaaacttccagtttaaatgtaaccgtaagtgcagcaactcttagttcaattgcagtt

actcctgcaaactcaagttatcctcttggtaaggtccaacaatacacagcaataggaaca

tacagcaatcagtccactcaagatttaacaaatcaggtttcctgggcatctctaaacact

tccgttgctacaatcgataattctgcatcctccaaaggccttcttactactcaatcgacc

ggttcagcgaacatcacagcaacgttaggcggaattactgggcagacacaagtaaatgtc

acctctgcagttcttactagtattacgatcactcctgcaaacccaagtgtagccaatgga

agaacattatatcttacagccaccggagttttctcagatggtacagtttctgacattacg

agccaagtaacgtggtccagttctttaacaagtgtagctactgcggataactccgccggt

ttatctggaagaattactggagtcggggtcggtagcacaaatatcaccgctgccatcgga

ggagtcgatattacaatttccttaaatgttaccaacgccactttagaatcgattcaagtg

gtttccgattcgcattcgatcgctcgaggtacatctacgtttgtacaagcaatcggaatc

tattcggatggttcttctcaaaacataagcgatcaagtcgcttggaacagctctaattct

tcaatattacagatctctaatttaaatgcagtccccaaaagagaaatacaatctccttct

tctggaagtttaggtacggcaaggattaccgcaactttagaagcaatctccgcatatacc

gacatttcggttaatgcagcaaccttagtttccatcgaagtatcacccacaaatccttcg

gttgctgctggccttactatttcttttacggcaaccggagtttataccgatggaagtaat

cagaaccttacttctcaagtaacttggaattcttccaacacgaatagggctacaatcagc

aacgcaaatggaacccaaggaatcgctttgggttcttccgccggaactacgaacatatcc

gcaacgttaggcgcagttacttcctccgctaccactcttacagtcacaaacgctgtttta

aattcgatcacgattactccatctattccttccatagcagtaggaagaagtttaaatctt

accgcaaccggaacttattctgatggaagtaatcaagacttgactacctccgtcgcttgg

acaagtgcggattcttccattgcttccgtagacaatgcttcgggtagacagggacaggca

acaggtgttacacaaggtaatactcaaatcagctctacgttaggcggaatttcttcaaca

atcagttttactgtaaatgctgcagttttagactcaattcaagtaactctggaagattct

cccattgcaaaaggaacttttacaagagcaatagcaacaggtgttttttcagatggaagc

aacttgaacattagtgaccaagttgtttgggatagttcacaaacaaacgtgattcaacta

ggagttttagaaaccggtcctaaaaagaaactgatgaattctcctgcaaacggaaacagt

actactggaacttcaagaatcactgcaacgttaggaggtgtaagcggatacgccgatctt

acagtaatcgctccaagtttaaccagtattcaaatcgatccaacacatccgagtgttgcc

aacggtctgactcaaaactttactgcaaccggagtttattcggatggtagcaatcaaaat

ctgaccgactccgttacttgggcttcttctaatcctgcagttgccacaatcagcaacgct

tccggaaccaacggtaaagcaaccactcttcaaaccggatctacaaatatcagcgcgagc

ctgggtaccgttacttccgatccaagtgtactaacggttacaaacgcaaccttaacaagc

atcacaatcgctcccacctcttctttcaacatcgcaaaagggttaaatcaagacttcgta

gcgacaggttattatacggatggctcgtctagggatttgactacccaagtcacttggaat

tcttccaatgtttcagtcgctactatcagcaatgcaaacggaactcaaggaagaatggcc

gcagttgatactggttctacaaatatctccgcatcgttaggaggaacatctagtccaaac

acaaatgtaaccgttacgtctgcggttctgaattcgatccaaatctctccagcagacatt

agtgtagccaaaggaaacaccaaggtatacaccgcgatcggagtatattcagattttagc

acgttagacgttacttctcaggttacttggacttcttccagtgtttcggttgctaccatc

agtaacgcaagtggtcacgaaggtttcgctacaactgtaggcacaggtacctccacaatt

accgccactcttggggggatttccaattctacgagtttaacggttacagccgccgtattg

gtttccctttcagtaggtcctaccaatagttttgtttatatgacacaaactaaaaatttt

acggctacaggaacatattctgatggaacaatgcaggatcttacaactcaagttacttgg

acctcttccgatacaaccttgggaacaatcagcaactcttttggaatagaaggcagggct

acaggaattgctgcaggtgccgtgacgctcacagcgactttgggaagtatcagtggaaac

acctctttgacggtaatctttttagatacgatcccacctacggtcacaaacgtagtcgct

ttgactcctactactgtaagaattacgtattccgaaaacgtaaacgaaatacaggcaaaa

atcgcagccaattacaaattagctcttacatcttctgtaactggaagttgttcagataac

agtaattttacttctacctcttctgtgattactgtttcctcagtaagtggaagcggatct

gtgttcgttttaactctaggttcttcacaaacatctaacgcaccttatacgattttagtg

aataaattgggagtacaagatctttctacaactccaaacaatttgggttgtgcaaactat

ggagattttttaggacaagaacaaatcaagattgtatctgcttcttgtgcaaattccaat

tctgtgattttgaacttctctaaggctcctaaatctggaaataatatcaccggatccgca

gaatgtaccggttctacagaatgttctaaccgttataaaatttccggagcaagcgatctg

ggaacgattaacagcgtgaaggtgttagatggaattgtttgtaacggagcgactgcggac

tctgcaaaagtatgtgtagtacataatttagtacaaaccggagcacaatatacgatcatc

accgcggactccgtagacggagacggatttgacaactcaagttggggatcaatccgaaat

tctttagatacggagaatcttcaatcttctccaagagatagagcttcctttttaggatgt

ggaacttctccggtcaactttgcagacggaccaatttctatagatccaaactcatccacg

tttggttatttaatcgattttaactctaagatctactcaggaccaaataattccggaaac

ggagcactccgattcgcctacgacggaagtattccagaatcagttcaattctcctttgaa

aaagatacaaccgttcaagatggtgatgcgactaacgtaagttctaacttagcttcttct

agagaaaattcgatctcagttccgccttatgtgacactaggacattccggatgtactaca

aataatggaactctttctttaggatgtggtccggataacgaaaacggaagaggtgtgttt

gcaactggaattctttccagcgcttcctatctatttgttgcaggtgcaaaaaccatagcg

gacggactgggacaatacttattcgattatctgtattactctgcagacccctcttctaat

atgagttacaaatatatagatctaggatcgatcactggaactttaaccgcaggaacttct

tctctaactgtgcttaacaatagagtgtttgcaggttttgcaaaatcaagcaacgacgga

atcggattattcggaggactcaacgcacccgatttcggatttgtaacgtttaactctgcg

gattcaggaactggattttgtactccgggttctaattgtgacgcgttcgatggaaccaaa

ggaaaaagaattcgaatcgacttccttccttacttcggaggaccgtctactggtttatta

ggaattaataataatgcacatccaaactgggcgtattatatcggagtagattccatgttc

gtatttaaaaaccgtatctacgctgcaaacggaggattacactcagtaggacataacggt

tccataatacgttctacaactacagatccaactgcggcttgtaccggaccagattcttgt

tctaattgggtagaaattggacctagaaccaatacaaaatggcataatagtcccacaaac

aactggttctctctggaattaaatcaattttataatctgattccgggagataaggcgttt

gcacaatttgctgaattcaacaataacctttatgtaactagaaccatttgtgttcaaagt

tctcaggcaactggaatcagaaccagtccaggaacggtagcgggatgtactgacggaacg

actacaaatcgaagagcacaactttggaaatgtgatccaactatttcaggaaacacaagt

gaatgtgacgcagcggattggtccgtggtgggagacgacggaaccggaatcacaaacatg

ggagattctacaaaccgaacgatcaccatggtgatgaaaaacggatcctatctttacata

ggatacgataatccaaacggaatcagaatttatagaaccaatgtagccaatccaggatca

tcctctgcgtcttggagtcaaatcgcgggcaacggtctaacagattcaactaacgtacaa

caaatttactctgcggtatccgtaccttcgggaagtatcaattatatttacgtaagcgta

ggaaaaagtaacgttccagttcggacgtatcgccaacaaaactaa

>fig|29507.8.peg.3299 surface protein Lk90-like protein [Leptospira kirschneri FMAS_PN5]

atgactaagcatatcaacaaactcagagataaaaaaacgcggtcttttcttcagtttact

tttgttttttttctaacattcaatttattttttttggaaagttgcgcggcttggccgatt

ttttcaggcgctccgggtttgttagcaggtaaaaaaggtggggcaaacaattcactttgg

atgctttttttaggagtaaacaacccacttgaatcggaaccatccgagacagagttagat

cggatcgaaatttccgtaccaaattcaagttttgctcgaggtaccactttacatctaaac

gccaccgccatctacaaagacaatactcatcgagatgtttcctcggaaggatcctggtcc

tctacggattcgagtattctcaagctattaacacaatctcaattcaaaggaatgaatcta

ggttctggaaatgtaaacgtatcctttcaaggaaaaaatgcaaccacaacgttaaccgtc

acatctgctgttttgtccgatttaaccgtaacttgtgtaaatcaaggtagtccattacct

gttggaatcgatcgtcaatgtaaattggaaggaattttttcagacggtagcactcaggtt

ttaacttccgatccaagtgcgtcttggaacataattcaatcttctatcgcaggtgtaaac

acaacaggtttagtttccgggctttctcctggcagcacttctattactagttcctatgga

agcaaaacttccagtttaaatgtaaccgtaagtgcagcaactcttagttcaattgcagtt

actcctgcaaactcaagttatcctcttggtaaggtccaacaatacacagcaataggaaca

tacagcaatcagtccactcaagatttaacaaatcaggtttcctgggcatctctaaacact

tcagttgcgacaatcgataattctgcatcctccaaaggccttcttactactcaatcgacc

ggttcagcgaacatcaccgcaacgttaggcggaattactgggcaaacacaagtaaatgtc

acctctgcagttcttactagtattacgatcactcctgcaaatccaagtatagccaatgga

agaacattatatcttacagccaccggggttttctcagatggtacagtttctgacattacg

agccaagtaacgtggtccagttctttaacaagtgtagctactgcggataactccgccggt

ttatctggaagaatttctggagtcggggtcggtagcacaaatatcaccgctgccatcgga

ggaatcgatattacaatttccttaaatgttaccaacgctactttagaatcgattcaagtg

gtttccgattcgcattcgatcgcccgaggtacatctacgtttgtacaagcaatcggagtc

tattcagacggttcttctcaaaacataagcgatcaagtcgcttggaacagctctaattct

ttaatattacagatctctaatttaaatgcagtccccaaaagagaaatacaatccccttct

tccggaggtttaggtacggcaaggattaccgcaactttagaagcaatctccgcatatacc

gacatctcggttaatgcagcaaccttagtttccatcgaagtatcacccacaaatccttcg

gttgctgctggccttactattccttttacggcaaccggagtttataccgatggaagtaat

cagaaccttacttctcaagtaacttggaattcttccaacacgaatagggctacaatcagc

aacgcaaatggaacccaaggaatcgctttgggttcttccgccggaactacgaacatatcc

gcaacgttaggcgcagttacttcctccgctaccactcttacagtcacaaacgctgtttta

aattcgatcacgattactccatctattccttccatagtagtaggaagaagtttaaatctt

accgcaaccggaacttattctgatggaagtaatcaagacttgactacctccgtcgcttgg

acaagtgcggattcttccattgcttccgtagacaatgcttcgggtagacagggacaggca

acaggtgttacacaaggtaatactcaaatcagctctacgttaggcggagtttcttcaaca

atcagttttactgtaaatgctgcagttttagactcaattcaagtaactctggaagattct

cccattgcaaaaggaacttttacaagagcaatagcaacaggtgttttttcagatggaagc

aacttgaacattagtgaccaagttgtttgggatagttcacaaacaaacgtgattcaacta

ggagttttagaaaccggtcctaaaaagaaactgatgaattctcctgcaaacggaaacagt

actactggaacttcaagaatcactgcaacgttaggaggtgtaagcggatacgccgatctt

acagtaatcgctccaagtttaaccagtattcaaatcgatccaacacatccgagtgttgcc

aacggtctgactcaaaactttactgcaaccggagtttattcggatggtagcaatcaaaat

ctgaccgactccgttacttgggcttcttctaatcctgcagttgccacaatcagcaacgct

tccggaaccaacggtaaagcaaccactcttcaaaccggatctacaaatatcagcgcgagc

ctaggcgcagttacttccgatccaagtgtactaacgattacaaacgcaaccttaacaaac

atcacaatcgctcccacctcttctttcaacatcgcaaaagggttaaatcaagacttcgta

gcgacaggttattatacggatggctcgtctagggatttgactacccaagtcacttggaat

tcttccaatgcttcagtctctactatcagcaatgcaaacggaactcaaggaagaatggcc

gcagttgatactggttctacgaatatctccgcatcgttaggaggaacatctagtccaaac

acaaatgtaaccgttacgtctgcggttctgaattcgatccaaatctctccagcagacatt

agtgtagccaaaggaaacaccaaggtatacaccgcgatcggagtatattcagattttagc

acgttagacgttacttctcaagttacttggacttcttccagtgtttcggttgctaccatc

agtaacgcaagtggtcacgaaggtttcgctacaactgtaggcacaggtacctccacaatt

accgccactcttggggggatttccaattctacgagtttaacggttacagccgccgtattg

gtttcactttcagtaggtcctaccaatagttttgtttatatgacacaaactaaaaatttt

acggctacaggaacatattctgatggaacaatgcaggatcttacaactcaagttacttgg

acctcttccgatacaaccttgggaacaatcagcaactcttttggaatagaaggcagggct

acaggaattgctgcaggtgccgtgacgctcacagcgactttgggaagtatcagtggaaac

acctctttgacggtaatctttttagatacgatcccgcctacggtcacaaacgtagtcgct

ttgactcctactactgtaagaattacgtattccgaaaacgtaaacgaaatacaggcaaaa

atcgcggccaattacaaactggctcttacatcttctgtaactggaagttgttcagataac

agtaattttacttctacctcttctgtgattactgtttcctcagtaagtggaagcggatct

gtgttcgttttaactctaggttcttcacaaacatctaacgcaccttatacgattttagtg

aataaattgggagtacaagatctttctacaactccaaacaatttgggttgtgcaaactat

ggagactttttaggacaagaacaaatcaagattgtatctgcttcttgtgcaaattccaat

tctgtgattttgaacttctctaaggctcctaaatctggaaataatatcaccggatccgca

gaatgtaccggttctacagaatgttctaaccgttataaaatttccggagcaagcgatctg

ggaacgattaacagcgtgaaggtgttagatggaatcgtttgtaacggagcgactgcggac

tctgcaaaagtatgcgtagtacataatttagtacaaaccggagcacaatatacgatcatc

accgcagactccgtagacggagacggatttgacaactcaagttggggatcaatccgaaat

tctttagatacggagaatcttcaatcttctccaagagatagagcttcctttttaggatgt

ggaacttctccggtcaactttgcagacggaccaatttctatagatccaaactcatccacg

tttggttatttaatcgattttaactctaagatctactcaggaccaaataattccggaaac

ggagcactccgattcgcctacgacggaagtattccagaatcagttcaattctcctttgaa

aaagatacaaccgttcaagatggtgatgcgactaacgtaagttctaacttagcttcttct

agagaaaattcgatctcagttccgccttatgtgacactaggacattccggatgtactaca

aataatggaactctttctttaggatgtggtccggataacgaaaacggaagaggtgtgttt

gcaactggaattctttccagcgcttcctatctatttgttgcaggtgcaaaaaccatagcg

gacggactgggacaatacttattcgattatctgtattactctgcagacccctcttctaat

atgagttacaaatatatagatctaggatcgatcactggaactttaaccgcaggaacttct

tctctaactgtgcttaacaatagagtgtttgcaggttttgcaaaatcaagcaacgacgga

atcggattattcggaggactcaacgcacccgatttcggatttgtaacgtttaactctgcg

gattcaggaactggattttgtactccgggttctaattgtgacgcgttcgatggaaccaaa

ggaaaaagaattcgaatcgacttccttccttacttcggaggaccgtctactggtttatta

ggaattaataataatgcacatccaaactgggcgtattatatcggagtagattccatgttc

gtatttaaaaaccgtatctacgctgcaaacggaggattacactcagtaggacataacggt

tccataatacgttctacaactacagatccaactgcggcttgtaccggaccagattcttgt

tctaattgggtagaaattggacctagaaccaatacaaaatggcataatagtcccacaaac

aactggttctctctggaattaaatcaattttataatctgattccgggagataaggcgttt

gcacaatttgctgaattcaacaataacctttatgtaactagaaccatttgtgttcaaagt

tctcaggcaactggaatcagaaccagtccaggaacggtagcgggatgtactgacggaacg

actacaaatcgaagagcacaactttggaaatgtgatccaactatttcgggaaacacaagt

gaatgtgacgcagcggattggtccgtggtgggagacgacggaagcggaatcacaaacatg

ggagattctacaaaccgaacgatcaccatggtgatgaaaaacggatcctatctttacata

ggatacgataatccaaacggaatcagaatttatagaaccaatgtagccaatccaggatca

tcctctgcgtcttggagtcaaatcgcgggcaacggtctaacagattcaactaacgtacaa

caaatttactctgcggtatccgtaccttcgggaagtatcaattatatttacgtaagcgta

ggaaaaagtaacgttccagttcggacgtatcgccaacaaaactaa

>fig|28184.25.peg.365 surface protein Lk90-like protein [Leptospira weilii FMAS_RT1]

atgttggtttttgagatgggttctacacaatcttcaggctctgcaaatgtcacggccact

ttaggcgcgattaccggtcaaacccaggttaacgttacctcggcggttcttacaagtatt

acgattactcctgccaatccaagcgttgctaacggaagaactttgtttttgaccgcaact

ggaattttttcggatggtacggcttccgatatcacaaaccaagtaacttggtccagttct

ttggcgagtgtcgctactgcggataactccgcgggtttatccggtagaattacaggaatc

ggggtcggtactacaaatatcaccgcggggatcggaggagtggataatactctttcttta

agcgtcacgaacgccgttttagaatcggttcaggtggtttccgattcttcttcgattgcc

agaggcacgtccacgttcgttcaggcgatcggagtctattcggacggttcttctcagaac

attagcgaccaggtcgcttggagcagttctaattcttccgttttgcaaatcgccaatttg

aatgcgattccgaaacgagaagtgcaatccccttcttccggcggttttggtacggcaagg

atcacggccactttggaagcgatttcctcgcatacggatatctcagtaactgcggcaact

ttaatttcgcttgaagtctctcccacaaatccttcggttgcagctggtcttacggttcct

ttcactgccacaggagtttatacggatggcagcaatcagaatttgacttctcaagcgact

tggaattcttctaacacaaatcgagccacgatcagcaacgcctcgggttccgaaggaatc

gccttaggttcttccgccggaactacgaatatttctgccacgttaggtgccattacttcc

tcctctacgactctcactgtcacaaatgcggttttaaattcgatcacgatcaccccggct

cttccttcgattgcaggtggaagaagtttgaatcttaccgcgactggagcttattcggat

ggaaatacccaggatttgactacttccgttgcctggacaagtgcggattcttccatcgct

tccgtagataacgctgcgggcagacagggacaaacaaccggtgtcgcacagggtacgact

caaatcagcgccttgttgggaggagtttccgctacgatcaattttacggtgaccgccgcg

gttttggattccattcaggttaccttggaagattctccgattgcgaaaggtacgtttacc

agggcgatcgcgaccggtgttttttccgacggtagcaatttgaatatcagcgatcaggtc

gtttgggatagctctcaaacaaatgtgatccaacttggaatcttagaagccggtcctaaa

aagaaactgatgaattctcccgcaaacggaagtagtaccacgggaacttcaagaatcact

gcaactctcggtggggtgagcggatttgccgaccttaccgtaattgctccgaacttggtc

agcattcagatcgatcccactcatccgaacgttgccaacggtttgtctcagaactttact

gctacgggtgtttattcggacggtagcaatcagaatctgaccgattccgtgacttgggct

tcttccaatccggctattgcgacgatcagtaacgcttcgggatcgaacggtaaggcgaca

atgcttcaaacgggatcgactaatatcagcgcgagtttgggcccggtcacttccgatccg

agtgttcttacggttacaagcgcgactttaacgagcatcacgatcgctccgacttcttct

ttcaatatcgcgaaaggattgaatcaaaactttgtagcgacgggttattatacggacggt

tcatccagagatctgacgtctcaggtgacttggacttcttccaatacttccactgctatg

atcagcaacgcgagcgggactcaaggaaaaatgacagcggtcgatacgggctctactaac

atttcggcgtctttaggaggaacgtcgagtgcgaatacgaacgtaacagtgacggcggct

gttttaaattcgattcaggtttcgcccgctgatatcagcgtggcaaaaggaaacacaaag

gcttacgctgcgatcggagtgtattcggattctacaacgttagacgtcacgtctcaggtg

acttggacttcttccaatacttccattgctacgatcagcaatgcgagcggtcacgaaggt

ttggctaccaccgtttctgccggaacgaccacgattaccgcaactctgggagcggtttcc

aattccacaagcctgaccgttacggccgcagtactggtttccctttcggtgggccctacg

aatagctttgtttatatgacacaaacgaagtattttacggctaccggaacatactccgat

ggaacgatgcaggacctgactacccaagtgacttggacttcttccgacacgactctggga

acggtgagcaacgctttcggaacggagggtaaggccacgggaattgcggccggagccgtg

acgatcactgccactctgggaagcatcagcggaaatacttctttgtccgtgatctttttg

gatacggttgctccgacggttacgaacgtagtggctttgactcctactacggtgagaatc

acgtattcggaaaacgtaaacgagattcaggctaaaactgcggccaattataaattagct

cttacgtcggctgtgagtggaagttgttcggataacagcaactttacttctacttcttct

gtgattactgtctcttcagtgagcggaagcggatccgtattcgttttaacgctcgggtcc

tctcaaacgtctaacgctccttatacgattctagtcaataagtcgggggtacaggacctt

tccactagtccgaacaatttgggttgtgcgaactatggtgacttcttaggacaggaacag

atcaaaatcgtttccgcttcctgcgcgaattccagctctgtgatcctaaatttttccaag

gctcctaagtccggaaataacatttcaggttctgcggaatgtaccggttctacggaatgt

gcaagccgttacaaaattgccggtgcgagcgatttgggaacgatcaacagcgctaagacg

ctggatggaattgtttgtaacggagcgacggccgattccgcaaaggtttgtgtcgtacac

aatcttgtgcaaacgggcgcacaatatactatcattgccgcggattccgcggacggggat

gggttcgacaacgcaagttggggatcaattcgaaattctttggatacggagaatcttcaa

tcttctccgagagacagggcttccttcctaggatgtggaacttctcccgtaaactttgcg

gatggaccgatttccatcgatccgaattcgtccacgttcgggtatctcatggatttcaac

tctaagatttattcgggaccgaataattccggaaacggggcactcaggttcgcttatgac

ggaagcgctcccgaatcggttcaattctcctttgaaaaggatacgaccgctcaggatggt

gatccgacaaatgtgagttcgaatatcgcttcttctcgggagaattcgatcgcgattccg

ccttacgtaactctgggacattccggatgtaccccaaacaacggaactctttctcttgga

tgtggtccggataacgaaagtggaagaggggcgttcgttacgggaattctttccagcgta

tcttatctttttgtcgcaggtgcgagaaccgtagcggacgggctcggacaatactttttc

gattatctgtattattccgcagatccttcttccaacatgagtttcaaatacatagacctt

ggctcgatcacggggactttgaccgctggaacttcttcgctgacggttcttaacaataga

gtgtttgccggttttgcgaaatccagcaacgacggagtcgggttgttcggaggactaaac

gcacctgatttcggatttgtaaccttcaactctgcggattccggaactggattttgtact

cccggttccaactgtgatgcgttcgacggaaccaaaggcaaaagaatccggatcgatttc

cttccttacttcggaggaccgtccacgggtttattaggactcaataataatgcgcatccg

aactggggatattatatcggagtcgattctatgttcgtattcaaaaatcgtatctacgcc

gcaaacggggggttacacgcggtagggcataacggatctatcatacgttctaccacggcc

gatcctacggcggcttgtacgggtcccgattcttgttcgaactgggtggagatcgggcca

agaaccaatacgaaatggcataacagcccgacgaacaactggttctctttggagttgaat

caattttacaacttgattcccggggacaaagcttttgcacaattcgccgaattcaacaac

aacctttacgtgacgagaactgtctgcgttcaaagttctcaggcgatcggaatcagaacg

agcgcgggaaccgtcgccggatgtacggacggaacaactacgaaccgaagggcgcaactc

tggaagtgtgatcctacgatttcgggaaacacgagcgagtgcgacgcggcggattggtcc

gtggtgggtgatgacggaaccggaatcacaaacatgggagattcgaccaaccggacgatt

acgatggtgatgaaaaacggatcttatctttacgtcggatacgataattcgaacggaatc

agaatttacagaactaacgtagccaatcccggatcgtcctccgcgtcttggagccagatc

gcggggaacgggctcacggacgcgacgaacgtacaacaaatctactcggcggtttccgta

ccttcgggaagtatcaattatatctacgtaagcgcgggaaaaagcggagtgccagttaga

acgtatcgtcagcagaactaa
