# Supplementary material for: Genomic analysis of human-infecting Leptospira borgpetersenii isolates in Sri Lanka: Expanded PF07598 gene family repertoire and less genome reduction than bovine isolates
Source: PLoS Negl Trop Dis. 2026 Mar 27;20(3):e0012540. doi: 10.1371/journal.pntd.0012540 (PMC13046281; doi:10.1371/journal.pntd.0012540)
Supplement: Attachment — Submitted filename: Reviewer Attachment for Manuscript Number PNTD-D-24-01330.docx [file pntd.0012540.s004.docx]

Reviewer Attachment for Manuscript Number PNTD-D-24-01330

"Genomic Analysis of Human-infecting *Leptospira borgpetersenii* isolates in Sri Lanka expanded PF07598 gene family repertoire, less overall genome reduction than bovine isolates"

1. Limited sampling:

The study is based on seven humans *Leptospira borgpetersenii* isolates from a specific region in Sri Lanka. While this data is valuable, the sample size is relatively small and geographically restricted. This limited sampling reduces the generalizability of the findings and raises questions about the representativeness of these isolates in other geographic regions and different human and animal populations.

**The discussion should more thoroughly address the need to validate these findings in a larger and more diverse sample, both geographically and in terms of host species. The impact of genetic variability under different environmental conditions and animal hosts could be better explored.**

2. Limited analysis of virulence and environmental factors

The authors highlight the presence of four VM proteins in the human isolates, suggesting a potentially important role in pathogenicity. However, the analysis of how these virulence factors translate into clinical phenotypes or relate to disease severity is insufficient. Furthermore, although the study suggests less genomic decay compared to bovine isolates, there is no direct investigation of how environmental factors influence pathogenicity.

**The discussion could benefit from a more detailed exploration of the functional implications of these VM proteins, including the need for future studies linking these genomic features to clinical manifestations of leptospirosis. Experimental or clinical studies should be suggested to validate the relevance of these proteins to virulence and transmission cycles.**

3. Inconsistencies in comparison between human and bovine isolates

While the study finds notable differences between human and bovine isolates in terms of genomic decay, pseudogenes, and insertion sequence (IS) elements, the comparison is based on a small number of bovine isolates. The absence of a broader diversity of animal isolates or the use of a more robust control group (with isolates from various animal species) limits the strength of the conclusions. Furthermore, there is no formal statistical analysis quantifying these differences, which could strengthen the findings.

**The comparison between human and bovine isolates should be discussed more cautiously, noting that the conclusions are based on limited data. The article could also suggest broader comparative studies that include isolates from multiple hosts to verify if these differences hold across different zoonotic environments.**

4. Lack of functional validation

Although the study uses whole-genome sequencing and bioinformatics to identify virulence genes, there is no functional validation of these findings. The absence of experimental studies that confirm the role of these proteins or genetic factors in Leptospira's life cycle or pathogenicity limits the applicability of the results.

**The authors could acknowledge the need for functional validation, such as gene expression experiments or animal models, to verify the relevance of the genomic findings. Without this validation, the conclusions regarding the importance of VM proteins and other virulence factors remain speculative.**

5. Limited impact on clinical practice and public health

While the study presents new information about the L. borgpetersenii genome, there is a gap in the discussion regarding how these findings could directly impact clinical practice or public health strategies, such as diagnosis, treatment, or outbreak control of leptospirosis.

The discussion could include a more detailed reflection on the practical implications of the findings. For example, how might the lower number of pseudogenes or the increase in VM proteins influence the development of new diagnostic tools, vaccines, or treatments? It would also be relevant to address how these discoveries could contribute to leptospirosis control in endemic areas.

**Conclusion**

While the study offers an important contribution, it is crucial for the authors to more explicitly address the limitations of sampling, host comparisons, the lack of functional validation, and the practical implications of the findings. Strengthening these areas in the discussion would enrich the manuscript, allowing readers to better understand the limitations and potential of the results presented.
