# Supplementary material for: Genomic analysis of human-infecting Leptospira borgpetersenii isolates in Sri Lanka: Expanded PF07598 gene family repertoire and less genome reduction than bovine isolates
Source: PLoS Negl Trop Dis. 2026 Mar 27;20(3):e0012540. doi: 10.1371/journal.pntd.0012540 (PMC13046281; doi:10.1371/journal.pntd.0012540)
Supplement: Attachment — Submitted filename: Response_to_Reviewers_table.docx [file pntd.0012540.s006.docx]

| **Comment (Reviewer 01)** | **Response** |
| --- | --- |
| Methods | |
| The methods used are appropriate for the genomic analysis, although more details must be provided for the Quality Control step during the initial processing of the sequencing data, for example. Additionally, it would important to provide more information about the version of the software. | This information were added to the methodology section. |
| **Comments (Reviewer 02)** |  |
| **Methods** |  |
| The authors solely used the Virulence Factor Database (VFDB) to identify virulence factors, which is a significant issue since the VFDB does not include Leptospira strains. This oversight limits the study's findings and prevents the identification of important virulence factors specific to Leptospira. To perform a reliable analysis, the authors should also conduct a manual search for previously identified Leptospira-specific virulence factors (e.g., Loa22, Lig, LipL32) in addition to using the VFDB. The lack of a thorough analysis raises concerns about the overall robustness of the study. | Manual searches were conducted using RAST seed viewer version 3. Those data were added to the table.The protein and DNA sequences will be included in supplementary files . The manual search description also added to the methodology section. |
| Only three of the seven new strains (PN1, AP8, and AP9) were used for the genomic alignment, all from Group 2. The absence of the other strains and the explanation for this omission should be clarified in the methods. | Figure was redrawn according the reviewer comments |
| While the authors referenced several published genomes for comparison (line 230), they need to include more details about these strains and their comparison in the methods section. | These details were given in method section |
| Additionally, the methods section contains a substantial amount of results (e.g., lines 195, 201). I suggest the authors to rewrite this section to be more direct and concise. | Those results were moved to the results section |
| **Comments (Reviewer 03)** |  |
| **Methods** |  |
| The methods used, such as whole genome sequencing and genomic variation analysis, are robust and appropriate for the objectives of the study. The Mauve alignment technique and the use of RAST for functional annotation are sound methodological choices. Data collection was well documented, with an adequate number of isolates analyzed to ensure statistically valid results. |  |
| **Comments (Reviewer 1)** | **Response** |
| **Results** |  |
| The results, in most parts, are well written and explored, although those from the functional analysis, using RAST, were poorly explored in the discussion, and it might be better explored, maybe comparing with other strains, from other hosts / serovars. | Thanks for the comment and well taken  Detail comparison was added to the discussion part  **Line 376-386**  Neela VK, Philip N, Raja P, Sekawi Z. Genomics data of L. borgpetersenii strain HP364 and L. weilii strain SC295 isolated from rodents captured from human leptospirosis outbreak areas in Selangor, Malaysia. Microbiology Resource Announcements. 2023 Dec 14;12(12):e00859-23. |
| Additionally, in line 291 the strains were classified as "non-serotypable", but more details (results or references) should be given to support this claim | Reference was added for this claim  **Line 309-310**  Jayasundara, D., Senavirathna, I., Warnasekara, J., Gamage, C., Siribaddana, S., Kularatne, S.A.M., Matthias, M., Mariet, J.F., Picardeau, M., Agampodi, S. and M. Vinetz, J., 2021. 12 Novel clonal groups of Leptospira infecting humans in multiple contrasting epidemiological contexts in Sri Lanka. *PLoS Neglected Tropical Diseases*, *15*(3), p.e0009272. |
|  |  |
| **Comments (Reviewer 2)** | **Response** |
| **Results** |  |
| The authors need to ensure that all tables and figures are submitted properly. The inclusion of several tables in the main body of the manuscript has caused significant formatting issues, making it difficult for me to fully analyze the data. | The number of table reduced according the comments table 4 and five were combined |
| The figure numbers are not match the references in the text. Please correct. | This was corrected |
| The authors should include the NCBI or UNIPRO IDs for the proteins listed in Tables 2 and 3. Additionally, Table 4 should be combined with Table 5 to improve the presentation of data. Consistency in the description of orthologs is also important; the authors need to ensure they use either LIC or LAI numbers consistently throughout the manuscript. | Table 4 and 5 were combined according to the instructions  Both LIC LA included in text |
| Since now PFAM data was integrated in INTERPRO, I suggest authors to also include the INTERPRO ID for the described domains. | IPR011455 id was added |
| While this information may be included in the missing portion of Table 5, the authors must clearly define the close orthologs (if LIC12339 or LIC12844) and which orthologs are present in previously published strains. This clarification is essential for understanding the comparative context of their findings. | These were added to the table |
| **Comments (Reviewer 3)** | **Response** |
| **Results** |  |
| The results are clear and offer detailed comparisons between human and bovine isolates of L. borgpetersenii. The identification of virulence factors, such as the expansion of VM proteins, and the presence of mobile elements provide a solid foundation for understanding the pathogenesis and transmission of the bacterium |  |
| **Comments (Reviewer 1)** | **Response** |
| **Conclusions** |  |
| The conclusions are aligned with the results in most parts, but it seems a bold statement to say that the ST 144 is the predominant ST in the dry zone of Sri Lanka, based only on 7 genomes. | I do agree with your comments the seven isolates doesn’t adequate for that  I have added the previous literature regarding circulating serovar in Sri Lanka which match with our finding  Line (336-338)  Karunanayake, L., Gamage, C.D., Gunasekara, C.P., De Silva, S., Izumiya, H., Morita, M., Muthusinghe, D.S., Yoshimatsu, K., Niloofa, R., Karunanayake, P. and Uluwattage, W., 2020. Multilocus sequence typing reveals diverse known and novel genotypes of Leptospira spp. circulating in Sri Lanka. *PLOS Neglected Tropical diseases*, *14*(8), p.e0008573. |
| **Comments (Reviewer 2)** | **Response** |
| **Conclusions** |  |
| While the search for VM proteins is a specific aim of the article, the authors miss a valuable opportunity by not exploring and discussing the existing literature on these proteins or providing a deeper analysis of their specific roles. It is essential to determine whether there is a way to distinguish the functions of the four orthologs and to identify which of these proteins are absent in previous genomes. Failing to engage with the relevant literature and provide this context significantly limits the depth and relevance of their findings. | Thank you for your insightful comments. Our study indeed suggests the potentially important role of the four VM proteins in human isolates, as highlighted. However, as noted, we did not investigate the relationship between these virulence factors and clinical phenotypes or disease severity in the current analysis. Further studies are needed to address these important questions. Additionally, while our findings indicate less genomic decay in human isolates compared to bovine isolates, investigating the influence of environmental factors on this decay was beyond the scope of this study and requires separate, focused research. We appreciate the opportunity to clarify these points and acknowledge the need for further investigations in these areas. |
| The authors mention the identification of a Type VI secretion system, but they only describe the identification of an ortholog of one component (clpV). Although orthologs for the inner-membrane component (VirB) have also been previously described, it does not seem reliable to conclude that Leptospira contains a Type VI secretion system based on their findings. The authors must clarify this point in the text to avoid any potential misunderstandings. | Description regarding typeVi secretory system was added and modified our observation  Reference  **Line (389-397)**  Coulthurst S. The Type VI secretion system: a versatile bacterial weapon. Microbiology. 2019 May;165(5):503-15. |
| **Comments (Reviewer 3)** | **Response** |
| **Conclusions** |  |
| The discussion adequately presents the implications of the findings. The conclusion that human L. borgpetersenii has fewer pseudogenes and IS elements than bovine isolates is consistent with the data presented. However, the question of the clinical applicability of these findings for diagnosis and treatment could be further explored. |  |
| **Additional comments (Reviwer1)** |  |
| Line 30: replace "severe disease" for "severe manifestations of the disease"  Line 88: replace "gram-negative" for "Gram-negative".  Line 105: According to NCBI Datasets, there are 966 genomes of Leptospira available at NCBI.  Line 159: add more information about the in-house quality control pipeline.  Line 159: use italic for latin terms such as "de novo".  Line 160: if the DnaA gene was used to adjust the phase of the genome, add this information here. | Correction was done highlighted in yellow  Correction was done highlighted in yellow  Corrected  More details were added  This was corrected  **DnaA gene** was not utilized to determine the starting point (or phase) for organizing the genome during assembly or annotation |
| **Additional comments (Reviwer2 )** |  |
| The authors should consider conducting a thorough proofreading and review of the manuscript for clarity, grammar, formatting, and overall quality before resubmission. Several sentences contain formatting or syntax issues that make them difficult for readers to follow. Here are a few examples:  Line 91: DALY value is not informed.  Line 120: VM is not defined (defined only in line 274).  Line 122: Syntax issue (While, In contrast).  Line 232: Number of tRNAs not informed.  Line 233: The item related to the value 226 is not informed.  Line 259-264: Text is confusing. It states that the central LCB on chromosome I differs significantly among isolates, yet also claims that FMAS_AP8 and JB197 have conserved regions throughout the genome,  Lines 304 and 328: Paragraph is repeated. | This was corrected  VM was defined according to the comments  This error was corrected  The number was added  Related value component was added  “FMAS_AP8 and JB197 have conserved regions throughout the genome”  was removed  It is not only about the LCB but also about the dimensions and locations.  The repeated part was removed from the discussion |
| **Additional comments (Reviwer3 )** |  |
