# Supplementary material for: Genomic analysis of human-infecting Leptospira borgpetersenii isolates in Sri Lanka: Expanded PF07598 gene family repertoire and less genome reduction than bovine isolates
Source: PLoS Negl Trop Dis. 2026 Mar 27;20(3):e0012540. doi: 10.1371/journal.pntd.0012540 (PMC13046281; doi:10.1371/journal.pntd.0012540)
Supplement: Attachment — Submitted filename: Response_Indika_R2_Decision Letter.docx [file pntd.0012540.s007.docx]

Response to Reviewer Comments:

Decision Letter

**Journal Requirements:**

1) We do not publish any copyright or trademark symbols that usually accompany proprietary names, eg ©,  ®, or TM  (e.g. next to drug or reagent names). Therefore please remove all instances of trademark/copyright symbols throughout the text, including:
- ® on page: 6.

**🡪Thank you. Fixed.**

2) Tables should not be uploaded as individual files. Please remove these files and include the Tables in your manuscript file as editable, cell-based objects. For more information about how to format tables, see our guidelines:
https://journals.plos.org/plosntds/s/tables

**🡪Thank you. Removed.**

3) We have noticed that you have uploaded Supporting Information files, but you have not included a list of legends. Please add a full list of legends for your Supporting Information files after the references list.

**🡪Thank you. Done.**

4) Please amend your detailed Financial Disclosure statement. This is published with the article. It must therefore be completed in full sentences and contain the exact wording you wish to be published.
1) State what role the funders took in the study. If the funders had no role in your study, please state: "The funders had no role in study design, data collection and analysis, decision to publish, or preparation of the manuscript."

**🡪Thank you. Done in the manuscript.**

5) Please modify your Competing Interests' statement in the online submission form and declare all competing interests beginning with the statement "I have read the journal's policy and the authors of this manuscript have the following competing interests:"
Note: If there are no competing interests to declare, please state "The authors have declared that no competing interests exist".

**🡪Thank you. Done.**

Reviewer #1: This manuscript reports the complete, circularized genomes of seven Leptospira borgpetersenii isolates from human leptospirosis patients in Sri Lanka . The study's findings challenge the established model that this species is defined by genome decay, a theory based on analyses of bovine-adapted strains . Comparative genomic analysis revealed these new human isolates, all belonging to ST144, are nearly identical and show significantly less genomic decay. They possess far fewer pseudogenes (136–146) and Insertion Sequence (IS) elements (46) compared to reference bovine strains like L550, JB197, and TC112, which have 248–400 pseudogenes and 116–121 IS elements. Additionally, these human isolates feature an expanded repertoire of four PF07598-encoded Virulence Modifying (VM) proteins, in contrast to the two or three homologs found in the bovine strains . The authors conclude that this ST144 lineage exhibits unique pathogenicity and that significant genome reduction is not a universal feature of L. borgpetersenii. In summary, the objectives are clear, and the material and methods used are appropriate.

**🡪Thank you. No response needed.**

Reviewer #2: (No Response)

**🡪Thank you. No response needed.**

Reviewer #3: The study presents a comparative genomic analysis of seven human-derived isolates of Leptospira borgpetersenii from Sri Lanka, contrasted with previously published animal-derived isolates. The objectives are clearly articulated, focusing on genome reduction, insertion elements (IS), and the PF07598 gene family. The study design is appropriate to address the stated aims.

**🡪Thank you. No response needed.**

The use of PacBio whole genome sequencing and de novo assembly is methodologically sound and of high quality. However, the Methods section lacks sufficient detail regarding raw data quality control procedures and the specific versions of the bioinformatic tools and databases employed, which limits reproducibility.

**🡪Thank you. We have modified the text and referenced our PLOS NTDS publication in which we described what was considered acceptable details** regarding the raw data quality control procedures and the specific versions of the bioinformatic tools and databases that we employed. These methods have been again vetted by our USDA bioinformatics collaborators listed in the authorship.

Manuscript: (lines 142ff):

As published,[33] SMRTbell libraries were generated and sequenced on a PacBio RS II system (Maryland Genomics, Institute for Genome Sciences, University of Maryland School of Medicine). A minimum of 800X read coverage was obtained for all seven isolates. Raw read data were preprocessed using an in-house developed quality control pipeline. The raw sequencing data (**Table S1** supplementary) was collected in FASTQ format using the PacBio sequencing technique. Prior to assembly, the data was quality-checked using the FastQC program to determine read quality, including the distribution of read lengths (see supplemental table for data details).[33] After the initial quality check, error correction was carried out, followed by trimming to remove low-quality elements and adapter sequences. The resulting trimmed reads were then used for *de novo* assembly with Canu 2.1 and were circularized using Circlator [17] (<http://sangerpathogens.github.io/circlator)>. Two overlapping contigs were recovered in all isolates after completion of the described workflow. The annotation was completed in all seven fully closed genomes using the NCBI Prokaryotic Genome Annotation Pipeline with default settings.

The use of Prokka and RAST for annotation is standard practice, and the implementation of MLST and cgMLST for isolate typing is appropriate. Nonetheless, the reliance on VFDB alone for virulence factor identification, without fully considering Leptospira-specific virulence genes, represents a limitation that should be addressed.

**🡪Thank you. We have noted this limitation (line number ).**

There are no ethical or regulatory concerns, as the work is based on genomic data obtained from previously published and ethically approved studies.

**🡪Thank you. No response needed.**

**Results**

-Does the analysis presented match the analysis plan?

-Are the results clearly and completely presented?

-Are the figures (Tables, Images) of sufficient quality for clarity?

Reviewer #1: Yes, the analysis match the analysis plan and the results are clearly presented.

**🡪Thank you. No response needed.**

Reviewer #2: (No Response)

**🡪Thank you. No response needed.**

Reviewer #3: The results are clearly presented and align with the stated objectives. Tables and figures effectively summarize genomic characteristics such as GC content, coding sequence number, and pseudogene counts. The identification of two distinct genomic clusters among the Sri Lankan isolates, together with the observation of reduced pseudogene and IS element content relative to bovine isolates, are important findings. The amplification of virulence-modifying (VM) proteins encoded by PF07598 family genes in human isolates represents a major contribution of this work. Figures illustrating genomic alignments and CRISPR/Cas arrangements are informative, but some require clearer labeling and improved legends to enhance comprehension. The results are consistent with the analysis plan and provide a coherent foundation for the conclusions.

**🡪Thank you. No response needed.**

**Conclusions**

-Are the conclusions supported by the data presented?

-Are the limitations of analysis clearly described?

-Do the authors discuss how these data can be helpful to advance our understanding of the topic under study?

-Is public health relevance addressed?

Reviewer #1: Yes.

**🡪Thank you. No response needed.**

Reviewer #2: (No Response)

**🡪Thank you. No response needed.**

Reviewer #3: The conclusions are generally well supported by the data and appropriately summarize the main findings of reduced genomic degradation and an expanded PF07598 gene family repertoire in human isolates. However, the assertion that ST144 is the predominant sequence type in the dry zone of Sri Lanka, based on only seven genomes, should be expressed with greater caution. The lack of functional validation for the identified VM proteins is a key limitation that should be more clearly emphasized. The discussion appropriately situates these findings within the broader context of Leptospira pathogenicity and evolution, but it would benefit from a deeper examination of CRISPR variability and its possible role in host adaptation.

The authors adequately address the public health relevance of their results, linking them to the epidemiological importance of human leptospirosis in Sri Lanka.

**🡪Thank you. No response needed.**

**Editorial and Data Presentation Modifications?**

Use this section for editorial suggestions as well as relatively minor modifications of existing data that would enhance clarity. If the only modifications needed are minor and/or editorial, you may wish to recommend “Minor Revision” or “Accept”.

Reviewer #1: Accept

**🡪Thank you. No response needed.**

Reviewer #2: (No Response)

**🡪Thank you. No response needed.**

Reviewer #3: The manuscript would benefit from improved organization of the Methods section to prevent the inclusion of results within it. All tables and figures should be clearly numbered and consistently referenced in the text. Figure captions and table legends should be expanded to make each visual element self-explanatory. The authors should also maintain consistency in ortholog nomenclature (e.g., LIC vs. LAI) to prevent confusion. Minor editorial corrections to improve clarity, readability, and precision of language are recommended. Overall, these are minor revisions that will strengthen presentation without affecting the scientific conclusions.

**🡪We have made extensive small editorial corrections, which can be seen in the manuscript with tracked changes.**

**Summary and General Comments**

Reviewer #1: The authors have made all the modifications / adjustments suggested by the reviewers and the manuscript seems ready for publication in it's current form

**🡪Thank you. No response needed.**

Reviewer #2: The manuscript by Senavirathna and colleagues presents a comprehensive genomic analysis of seven non-Hardjo Leptospira borgpetersenii isolates from human leptospirosis patients in Sri Lanka. The authors show that these isolates are less genomically decayed than previously reported Hardjo strains, with fewer pseudogenes and insertion sequence elements, and an expanded VM protein repertoire. This work provides novel insights into the genomic diversity and potential pathogenicity of the under-studied species L. borgpetersenii, making it highly suitable for publication in this journal. However, while the authors have addressed some points from the previous review, many suggestions remain overlooked, and the manuscript continues to present major formatting, syntax, and grammar issues that require careful attention. Despite the extensive list of issues to be addressed, they all appear to require only minor revisions.

1. Formatting issues: The manuscript was submitted in landscape orientation, lines were not justified, and the response to reviewers did not include line numbers. These issues made the manuscript very difficult to review.

**🡪Responses are detailed and refer to line number, and, more importantly, are marked in marked-up document. I prefer not to right-justify manuscripts and have not done so.**

2. Grammar and Syntax: Although some issues raised in my initial review have been properly addressed, many of the previous suggestions were not. In particular, as mentioned, the authors should carefully review the grammar and syntax throughout the manuscript. Several sentences previously noted for correction (e.g., Line 122: Syntax issue - While, In contrast) were overlooked. I listed several examples in the minor comments, but the issues might not be limited to those points.

**🡪Extensive revision of these details have been made and are shown in the revised manuscript provided with tracked changes.**

Manuscript: (lines 142ff):

As published,[33] SMRTbell libraries were generated and sequenced on a PacBio RS II system (Maryland Genomics, Institute for Genome Sciences, University of Maryland School of Medicine). A minimum of 800X read coverage was obtained for all seven isolates. Raw read data were preprocessed using an in-house developed quality control pipeline. The raw sequencing data (**Table S1** supplementary) was collected in FASTQ format using the PacBio sequencing technique. Prior to assembly, the data was quality-checked using the FastQC program to determine read quality, including the distribution of read lengths (see supplemental table for data details).[33] After the initial quality check, error correction was carried out, followed by trimming to remove low-quality elements and adapter sequences. The resulting trimmed reads were then used for *de novo* assembly with Canu 2.1 and were circularized using Circlator [17] (<http://sangerpathogens.github.io/circlator)>. Two overlapping contigs were recovered in all isolates after completion of the described workflow. The annotation was completed in all seven fully closed genomes using the NCBI Prokaryotic Genome Annotation Pipeline with default settings.

3. Reference genomes: Different reference genomes were used in different parts of the paper as comparison (e.g., Table 1 vs Table 4). While I appreciate the inclusion of several reference genomes, the analyses should be consistent, and the chosen references should be clearly described. It is also unclear where in the text the details about these reference genomes were added, as previously suggested.

4. Clarity of strain comparisons: Readers may struggle to follow the results when only the strain names are repeatedly given. I recommend distinguishing more clearly the results between "analyzed" and "reference" strains throughout the text.

**🡪Done, as seen in multiple places in revised manuscript with changes tracked.**

5. VM repertoire analysis: This is a major finding of the work but is not adequately addressed. While Table 4 lists the closest VM orthologs, would be interesting to know how these orthologs differ (e.g., identity/similarity between them). The authors should discuss the presence of 12 VM proteins in LIC and LAI (used as references) and explain the differences among them, to help interpret why all identified orthologs matched only two LIC VM orthologs (with one repeated).

**🡪Thank you for this comment. This is clarified with extensive rewriting in the manuscript and we have provided better references to the VM protein/PF05798 orthologs to clarify these relationships for the reader (lines 423, ff).**

6. Gene identifiers: The previous suggestion to standardize the use of LIC vs LAI gene ids has not been fully addressed. Both naming systems are still mixed in the text, making it hard to follow. The authors should select one format and use it consistently.

**🡪 Response. Done. We use the LIC nomenclature first, followed by LA nomenclature in parentheses for clarity.**

7. Inconsistent naming: Strains and sequence types are reported in different formats (e.g., ST144 vs ST 144, JB197 vs JB 197). This should be standardized across the text.

**🡪 Response. Done.**

All of the following have been fixed:

Line 26. Sentence is hard to follow. Why are only two values for insertion sequences (IS) described for three different strains?🡪this sentence was modified.
Line 69. Syntax: “..have, either in final or draft form, have..” ? 🡪rewritten
Line 72. Would be interesting to define the year that the first genome was published. 🡪2003, added reference.
Line 79. Syntax: “While… In contrast..” 🡪rewritten
Line 121. RAST should be defined earlier. 🡪it is fine as written
Line 129. Authors increased the number of strains included in the alignment, as suggested, but did not adjust the methods accordingly. 🡪rewritten
Line 149. Grammar: “were ranged”. 🡪rewritten
Line 156. (37) is not needed. 🡪fixed
Line 157. Need to better explain what the subsystems are. Do authors know which one is missing in FMAS_AP4? 🡪fixed
Line 158. The numbers for subsystems contradict the previous sentence. Does FMAS_AP2 have 170 or 225 predicted subsystems? 🡪fixed
Line 164. Even with authors properly defining and discussing ST114 in the discussion, I suggest briefly defining it in the results to facilitate reader understanding. The sentence is also confusing as it sounds that the ST114 was defined by the CRISPR-Cas finder tool.🡪clarified.
Line 183. Syntax: “However” is not needed. Review sentence structure.🡪fixed
Line 184. As per my major review suggestion, why is the piyasena strain only included as a reference later in the work? 🡪This is just how the narrative turned out.
Line 237. CRISPR needs to be defined earlier.🡪This is just how the narrative turned out.
Line 289. Ensure consistent use of orthologs/paralogs/homologs throughout the paragraph🡪This is how we would like to write this.
Table 1. Syntax: “were given in the table were generated🡪fixed
Table 4. Locus Tag header should be renamed to “Closest Ortholog in LIC/LAI.” 🡪fixed
Table 4. Footnote contains syntax issues: “is given to compared”. Revise for clarity. 🡪fixed

Reviewer #3: This manuscript provides a valuable genomic contribution to the understanding of Leptospira borgpetersenii in human isolates from Sri Lanka, offering new insights into virulence evolution and host adaptation. The work is original and scientifically significant, demonstrating that human isolates exhibit less genomic reduction and a broader PF07598 gene family repertoire compared to bovine isolates. The study employs a robust methodological framework, including high-coverage PacBio sequencing and comparative genomic analyses. However, reproducibility would be enhanced by providing detailed information on the bioinformatics pipeline, software versions, and quality control metrics. The interpretation of a complete Type VI secretion system based solely on the identification of ClpV orthologs should be revised to reflect greater caution.

**🡪 Response.** We have noted this limitation, along with a citation in the revised manuscript (lines 131, ff): A limitation of our approach is that we have not experimentally demonstrated the presence of a Type VI secretion system, similar to the limitation of our previous comparative genomic analysis publication [3].

**🡪Response. Detailed information on the bioinformatics pipelines has been provided (see above).**

Overall, the manuscript is of high quality, well contextualized within the current literature, and represents a meaningful contribution to the field of leptospiral genomics and neglected tropical diseases. I recommend acceptance after minor revision, addressing the methodological transparency, clarifying figure legends, and tempering interpretative statements that extend beyond the presented data.

**🡪Thank you. No response needed.**
